# Vascular regional analysis unveils differential responses to anti-angiogenic therapy in pancreatic xenografts through macroscopic photoacoustic imaging

**DOI:** 10.7150/thno.99361

**Published:** 2025-01-27

**Authors:** Allison Sweeney, Andrew Langley, Marvin Xavierselvan, Ronak T. Shethia, Patrick Solomon, Aayush Arora, Srivalleesha Mallidi

**Affiliations:** 1 Department of Biomedical Engineering, Tufts University, Medford, MA, United States.; 2 Wellman Center for Photomedicine, Massachusetts General Hospital, Boston, MA, United States.

**Keywords:** pancreatic cancer, angiogenesis, sunitinib, vascular density, photoacoustics, endogenous contrast, vascular normalization

## Abstract

**Background:** Amongst the various imaging techniques that provide surrogate tumor radiographic indications to aid in planning, monitoring, and predicting outcomes of therapy, ultrasound-guided photoacoustic imaging (US-PAI) is a promising non-ionizing modality based on endogenous blood (hemoglobin) and blood oxygen saturation (StO₂) contrast. Adaptation of US-PAI to the clinical realm requires macroscopic system configurations for adequate depth visualization.

**Methods:** Here we present a vascular regional analysis (VRA) methodology of obtaining areas of low and high vessel density regions within the tumor (LVD and HVD respectively) by frequency domain filtering of macroscopic PA images. In this work, we evaluated the various vascular and oxygenation profiles of different murine xenografts of pancreatic cancer (AsPC-1, MIA PaCa-2, and BxPC-3) that have varying levels of angiogenic potentials and investigated the effects of receptor tyrosine kinase inhibitor (sunitinib) on the tumor microvessel density and StO₂.

**Results:** The administration of sunitinib resulted in transient deoxygenation and reduction in vessel density within 72 h in two (AsPC-1 and MIA PaCa-2) of the three tumor types. Utilizing VRA, the regional change in StO_2_ (∆StO_2_) revealed the preferential targeting of sunitinib in LVD regions in only the AsPC-1 tumors. We also identified the presence of vascular normalization (validated through immunohistochemistry) in the sunitinib treated AsPC-1 tumors at day 8 post-treatment where a significant increases in HVD ∆StO_2_ (~20%) were seen following the 72-hour time point, indicative of improved vessel flow and functionality. Treated AsPC-1 vasculature displayed increased maturity and functionality compared to non-treated tumors on day 8, while these same metrics showed no conclusive evidence of vascular normalization in MIA PaCa-2 or BxPC-3 tumors.

**Conclusion:** Overall, VRA as a tool to monitor treatment response allowed us to identify time points of vascular remodeling, highlighting its ability to provide insights into the tumor microenvironment for sunitinib treatment and other anti-angiogenic therapies.

## Introduction

Pancreatic cancer (PC) is the third leading cause of cancer-related deaths in the United States with a median survival time of 4.6 months and 5-year survival rate of 12% 1, 2. Surgical resection coupled with systemic chemotherapy is currently the only curative treatment option for patients 3. This poor prognosis can be attributed in part to the asymptomatic nature of PC that leads to late-stage detection, leaving only 10-20% of those diagnosed eligible for surgical resection 4. Borderline-resectable PC must rely on preoperative therapy to shrink tumors prior to resection to improve surgical outcomes 5, 6 and neoadjuvant chemotherapy has been shown to increase survival, improve the chance of a full resection, and reduce the frequency of positive margins following surgery 4, 7. Despite this, a considerable proportion of patients (~80%) with PC experience recurrence after undergoing resection and/or neoadjuvant chemotherapy, ultimately resulting in patient death 8, 9. The biological factors underpinning the aggressive and treatment-resistant nature of PC can in part be attributed to features of the pancreatic tumor microenvironment (TME) 10. The pancreatic TME is characterized by an abundance of stromal cells and extensive extracellular matrix, while remaining hypovascularized 10-12, resulting in a severely hypoxic TME which significantly influences tumor metabolism, therapeutic resistance, and angiogenesis 10, 12, 13. Angiogenesis, the formation of new blood vessels, is a crucial element in the growth and spread of solid tumors. The irregular shape and function of tumor vasculature may result in decreased blood flow and oxygenation, impeding the delivery of therapeutics to the tumor site 14-16.

Angiogenesis inhibitors are a family of therapeutics that act by suppressing the development of new blood vessels and recent research has shown that antiangiogenic treatment may be tailored to normalize tumor vasculature 17-19. Vascular normalization employs modest doses with brief treatment durations to ameliorate the structural and functional irregularities of tumor blood vessels and sensitize tumor tissue to conventional therapy 17, 20-22. Enhancing the uniformity of functional vascular density and improved configuration of arteries can lead to a decrease in regions of hypoxia and acidosis 17, 23-25. In preclinical models, vascular normalization via anti-angiogenic therapies has been demonstrated to boost tumor blood supply and oxygenation 26-28, reduce metastatic burden 29, 30, and enhance the efficacy of ionizing radiation 31-34, chemotherapy 35-38, and immunotherapy 39-42. The majority of antiangiogenic drugs target the vascular endothelial growth factor (VEGF) pathway, either by inhibiting VEGF-A with neutralizing antibodies or by blocking the VEGF-receptors (VEGFRs) with tyrosine kinase inhibitors (TKIs) 43-45. Sunitinib is a multi-targeted TKI which utilizes the latter approach, inhibiting the activity of a number of tyrosine kinases, such as VEGFRs, platelet-derived growth factor receptors (PDGFRs), and stem cell factor receptors (KIT) 46, 47.

By inhibiting VEGFR, sunitinib limits endothelial cell proliferation and migration, ultimately decreasing overall vascular density in the tumor 48, 49. The more organized and structurally stable vessels that may result from this process can enhance perfusion, but excessive inhibition can induce hypoxia and upregulate hypoxia inducible factors, complicating the tumor's vascular architecture 32, 47-69. The inhibition of PDGFR works synergistically with VEGFR inhibition and its inhibition by sunitinib leads to inadequate pericyte coverage. This lack of coverage can result in a more permeable and less functional vasculature, further impacting hemodynamics and potentially leading to compromised tumor oxygenation 59, 70-72. Sunitinib is authorized for the treatment of a variety of cancers, including renal cell carcinoma and gastrointestinal stromal tumors (GIST) such as PC 58, 65 Sunitinib remains the only targeted therapy currently approved for both GISTs and PCs, according to the NIH National Cancer Institute 73, highlighting its unique relevance in the context of this study. Much promise has been shown in administering sunitinib in combination with traditional treatments for PC as sunitinib has been demonstrated to make PCs more sensitive to radiation treatment *in vitro* and* in vivo* 66, 68. Studies on combinatorial treatment have shown that the co-administration of sunitinib with gemcitabine in orthotopic PC models and nab-paclitaxel in subcutaneous PC models enhanced survival and reduced tumor burden compared to monotherapy 55, 69.

Although rapid innovations in cancer therapeutics have allowed for more targeted destruction of solid malignancies, evaluating therapy response remains an obstacle, as there are limited endogenous radiographic indicators to aid in therapy planning and monitoring. Monitoring vascular structure and function is of particular interest, as these factors play a pivotal role in understanding therapy-induced changes in the TME 10, 12, 46. Research into the functional indicators of therapy response in PC is critical for giving timely, accurate feedback on treatment efficacy and developing strategies for personalized medicine. Ultrasound-guided photoacoustic (PA) imaging (US-PAI) has received a lot of attention in recent years due to its non-invasive capacity to provide spatially co-registered anatomical, functional, and molecular data of the TME using endogenous contrast. PAI combines optical excitation and acoustic detection to generate high-resolution images containing functional information about biological tissues 74, 75. PAI involves delivering nanosecond pulsed light into tissue, which is absorbed by chromophores and converted into heat causing thermoelastic expansion and contraction of the absorber and generation of acoustic waves detectable by US transducers 76, 77. The minimal scattering of acoustic waves in biological tissues allows this hybrid modality to reap the benefit of increased penetration depth compared to purely optical techniques 74, 78. Based on wavelength selection, US-PAI can display detailed functional and molecular information for a wide range of endogenous chromophores 79, 80. The absorption spectra of hemoglobin changes when bound to oxygen, allowing the use of multi-wavelength PAI to assess blood oxygen saturation (StO₂) and hemoglobin concentration (HbT) by independently measuring oxyhemoglobin (HbO_2_) and deoxyhemoglobin (Hb) distributions 81. Among approaches used to image vasculature, PAI stands out for its exceptional scalability, making it suitable for imaging in the micro- to macroscopic scales. PAI has shown to be a promising modality to evaluate and monitor response to anti-angiogenic therapies based on changes in vascular morphology and StO₂, which are strongly associated with tumor hypoxia, according to preclinical investigations in murine models 82-85.

In addition to StO₂, microvessel density (MVD) has shown a correlation with aggressiveness in a variety of malignancies 86-89. The emergence of tailored anti-angiogenic medication provides the possibility to use MVD analysis as both a prognostic and therapeutic marker. MVD can be measured with a variety of histological and *in vivo* imaging techniques, including PAI 89-92. To our knowledge, the use of PAI to measure MVD *in vivo* has been mostly limited to PA microscopy (acoustic and optical resolution) or mesoscopy 92-100. Microscopy techniques may attain far greater spatial resolution, but they are usually restricted to depths up to 1 mm 101. In PAI specifically, mesoscopy refers to depths from 1-5 mm, with a resolution in the range of a few to tens of microns 100, 101. In the push towards clinical translation of PAI, there comes the hurdle of balancing system resolution and penetration depth. Clinical imaging of tumors, specifically volumetric imaging, will require macroscopic configurations for adequate depth visualization. PA macroscopy encompasses depths exceeding 5 mm and offers resolution ranging from tens to hundreds of microns, in which individual microvessels cannot be resolved 102. This illuminates the need for a surrogate marker to classify relative MVD within a tumor using macroscopic PAI. Herein, we investigate the feasibility of a surrogate imaging marker for vascular density in PC xenografts treated with sunitinib. We have chosen to utilize sunitinib as a proof of concept therapy because it has been shown to preferentially target immature vasculature and can induce vascular normalization in PC 32, 49. We hypothesize that frequency domain filtering of macroscopic PA images will allow us to regionally classify high and low vascular density (HVD and LVD) areas and that our classification will show good agreement with the distribution of endothelial marker CD31. Utilizing vascular regional analysis (VRA) of treatment-induced StO₂ and HbT changes, we anticipate that sunitinib will preferentially reduce StO_2_ and HbT in LVD regions during early treatment time points, as sunitinib selectively prunes small vessels in PC 49. Additionally, given the advent of vascular normalization, we will show the feasibility of VRA as a tool for identifying key time points of vascular remodeling in low-resolution macroscopic PAI configurations.

## Materials and Methods

### Cell lines and animal models

All animal studies in this work were approved by Tufts University's Institutional Animal Care and Use Committee (IACUC). Male homozygous Foxn1^nu^ nude mice (The Jackson Laboratory) were subcutaneously injected with 5 million AsPC-1, MIA PaCa-2, or BxPC-3 cells in 100 µL of Matrigel (50 µL of Matrigel + 50 µL of phosphate-buffered saline (PBS) using a 28-gauge insulin syringe. All cells were obtained from the American Type Culture Collection and supplemented with 10% fetal bovine serum and 1% penicillin-streptomycin (100 U/ml). BxPC-3 and MIA PaCa-2 cells were cultured in RPMI-1640 (Roswell Park Memorial Institute), while AsPC-1 was cultured in DMEM (Dulbecco's Modified Eagle Medium) media. All cells were grown in a T-75 flask and maintained in a humidified incubator at 37 °C and 5% CO_2_. AsPC-1 and BxPC-3 cells were passaged 1-2 times each week, while MIA PaCa-2 cells were passaged 2-3 times per week.

### Sunitinib treatment

The sunitinib solution was prepared at a concentration of 20 mg/mL by dissolving 200 mg of sunitinib L-malate (Sigma Aldrich) in 1 mL of dimethyl sulfoxide and 9 mL of corn oil. The solution was repeatedly vortexed and placed in a 50 °C ultrasonic water bath for intervals of 2 min each until no visible lumps or particles were present. The sunitinib solution was stored in 4 °C. Mice were treated with 80 mg/kg per day with sunitinib or vehicle for 20 days via oral gavage. The treatment regimen began once tumors reached a volume of approximately 50-150 mm^3^. Mice were split into the following groups: Sunitinib group (MIA PaCa-2: n = 13; AsPC-1: n = 9; BxPC-3: n = 7) and no treatment (NT) group receiving 1x PBS (MIA PaCa-2: n = 11; AsPC-1: n = 8; BxPC-3: n = 5).

### Longitudinal photoacoustic imaging

**Figure 1:**
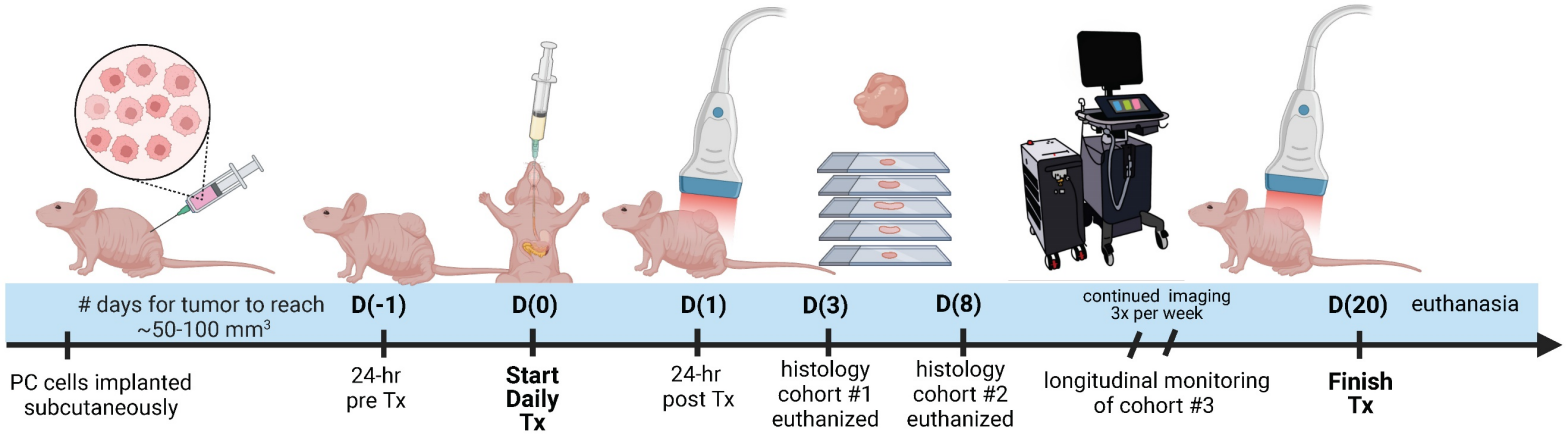
Study timeline describing the imaging, treatment, and histological examination of pancreatic tumor xenografts. D(0) represents Day 0, which is the day of the first treatment or vehicle administration. Cohort #1 was euthanized on D(3), cohort #2 was euthanized on D(8), and cohort #3 was monitored longitudinally up to D(20).

The experiment timeline is displayed in Figure 1 with image acquisition for each mouse starting once tumor volume reached a minimum of 50 mm^3^. Pre-treatment imaging was performed 24 h before administration of the first dose (Day -1: D(-1)). Imaging during the treatment period was performed at precisely 24 and 72 h (Day 1: D(1) and Day 3: D(3)) after the first administered dose and continued thrice weekly (Day 6 and beyond: D(6+)). On D(3) post-treatment, 3 tumor specimens were extracted to utilize for validation of the VRA algorithm, deemed cohort #1. On D(8) post-treatment, 12 specimens were extracted from cohort #2 and histologically evaluated for AsPC-1 and MIA PaCa-2, with 6 total tumors from each cell line (3 per treatment group) to assess proliferation, vascular maturity, and perfusion. Mice euthanized on D(8) were injected with Tomato Lectin (TL) conjugated with DyLight® 488 (DL-1174-1, Vector Laboratories) 5-7 min before euthanasia. Cohort #3 encompassed the remaining mice and was longitudinally monitored with US-PAI through the last day of the treatment regimen (D(20)).

The Vevo LAZR-X (Fujifilm, VisualSonics) was used to capture US and PA images, utilizing the MX250S linear array transducer with a 6 dB bandwidth of 15-30 MHz, central transmit frequency of 21 MHz, axial resolution of 75 µm, and lateral resolution of 165 µm. The transducer was coupled to an integrated 20 Hz tunable laser (680-970 nm) via fiber optic cables. Throughout each of the imaging sessions, gain (22 dB for US, 45 dB for PAI) and persistence (20 averages per frame) remained constant. The Vevo LAZR-X Oxy-Hemo mode (750 and 850 nm laser pulses) was used to generate the PA images.

The imaging workflow was performed as follows. During the imaging session, mice were sedated with isoflurane (2-3% induction, 1.5% maintenance) with 100% oxygen gas and placed on a heating pad with electrocardiogram (ECG) leads to monitor body temperature, heart rate, and breathing. To improve acoustic transmission between the transducer and the tumor during imaging, a bubble-free ultrasonic transmission gel (Aquasonic100 Ultrasonic Transmission Gel, Parker Laboratories, Inc.) was applied to the tumor. For each frame, an average of 20 images were captured at two wavelengths (750 and 850 nm), yielding a 2D PA image of tumor StO₂ in roughly 10 seconds. Each dual-wavelength 3D image of a tumor was acquired within 20 to 30 min, with the first imaging frame recorded at the back end of the mouse with each succeeding frame moving a 0.15 mm step toward the anterior.

### Fluence compensation

Fluence compensation was performed using Monte Carlo simulations utilizing the Monte Carlo eXtreme (MCX) package for MATLAB 103, 104 and the PHotoacoustic ANnotation TOolkit for MATLAB (PHANTOM) 105. Fluence compensation maps were generated for and applied to all PA images used in analysis. Briefly, 10 million photons (5 million from each fiber) were discharged from the light source toward the tissue volume. For every 0.1 mm^3^ voxel inside the tissue volume, the optical characteristics of absorption coefficient, scattering coefficient, anisotropy factor, and refractive index were assigned dependent upon the tissue type in that area. Different optical properties were assigned for skin, soft tissue, and tumor tissue, which are summarized in Table S1. The localization and labeling of these tissue types within the volume were done utilizing the co-registered US images. In the simulation, water served as the interface between the light source and the tissue, to represent the ultrasound gel used as an acoustic coupling layer between the transducer and tissue. Photon propagation was maintained for 5 ns, which corresponds to the width of a common laser pulse used in PAI. Non-reflective boundary conditions were used throughout the simulation. As the transducer moves throughout the tumor volume to obtain a 3D scan, separate simulations were done for each transducer location and compiled to generate the resulting fluence map. More detailed information on the simulation geometry and parameters used for Monte Carlo parameters can be obtained from Sweeney *et al.* 105.

### Oxygen saturation and hemoglobin imaging via multi-wavelength PAI and spectral unmixing

The wavelength (λ) and depth (z) dependent photoacoustic initial pressure produced by pulsed light stimulation of optical absorbers, assuming stress confinement, may be represented by EQ. 1.


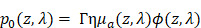

(1)

Approximated as constants, 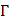 represents the Grüneisen parameter, and 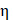
represents the fraction of absorbed light converted into heat. The non-constant 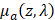 represents the optical absorption coefficient, and 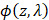 represents the light fluence. The absorption coefficient is simply the product of the molar extinction coefficient, 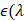), and concentration, 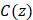, of a specific chromophore. In the near-infrared I (NIR I) wavelength range, the absorption of water and lipids is negligible compared to hemoglobin and the mice imaged in this study had insignificant skin pigmentation, allowing us to ignore the contribution of melanin. With the assumption that hemoglobin is the primary biological absorber being imaged, EQ. 1 can be re-written as EQ. 2.


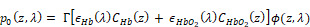

(2)

As the two wavelengths used in this study were 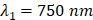 and 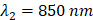, the molar concentrations of Hb (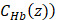
and HbO_2_ 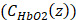 were obtained by solving EQ. 3 with the non-negative linear least squares method. The molar concentration values of Hb and HbO_2_ were input into EQ. 4 and EQ. 5 to calculate StO₂ and HbT respectively.


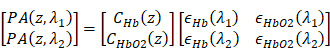

(3)


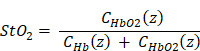

(4)


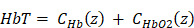

(5)

Where 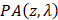 represents the fluence compensated PA image i.e. 
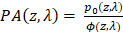
 .

### Image denoising

To improve the signal-to-noise ratio (SNR) of the StO₂ images, the HbT images were analyzed to find a noise threshold. The noise threshold of each image was calculated by taking the average HbT signal from two 50x50 pixel ROIs and averaging this across each frame in the volumetric image. The maximum value for each mouse was then averaged across all mice for all time points (Table S2) and rounded to the nearest ten-thousandth to produce a generalized threshold to be applied to all images. If the HbT value of a particular voxel in the full volumetric image was less than the specified threshold, the regions were considered to be avascular, and the corresponding pixel in the oxygen saturation matrix was set to zero and omitted from further analysis.

### Vascular regional analysis (VRA)

To segment the PA images into regions of HVD and LVD, frequency domain filtering was applied to the volumetric HbT images acquired from spectral unmixing as described in section 2.4 of the text. For clarity, no Fourier analysis was performed on pre-beamformed or single-wavelength PA volumes. Volumetric HbT images were normalized to a range of [0,1] and are denoted as I(x,y,z). A 3D Fast Fourier Transform (FFT) was performed on I(x,y,z) to get F(u,v,w) in the Fourier space (EQ. 6) and rearranged by shifting zero-frequency components from the edges to the center of the matrix.


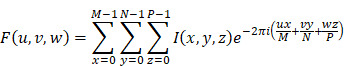

(6)

A Gaussian high pass filter (HP) was applied by elementwise multiplication with F to get the filtered volume (F'(u,v,w)) as shown in EQ. 7. The high pass filter was utilized to remove the low-frequency components of the image, which we hypothesized would contain signal corresponding to regions of low vascular density.


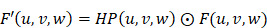

(7)


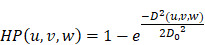

(7.1)


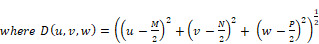
 , (7.2)



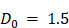



The 3D inverse Fourier transform was used to transform F' back into the spatial domain (EQ. 8) after rearranging the zero-frequency components back to the edges of the image to get the filtered volume (I'(x,y,z)). The values from the filtered volume, I', were not used in any data analysis but rather used specifically to segment the relative areas of high vascular density.


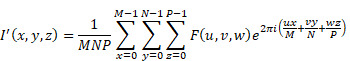

(8)

To regionally segment the tumor, the tumor mask was applied to I', which was then binarized using a modified Otsu thresholding method for log-normal (LN) distributions 106, 107 to obtain a mask where the foreground represents regions of HVD as shown in EQ. 9. We chose to apply a modified version of Otsu's method for LN distributions as the high-pass filtering step resulted in the HbT intensity distribution changing from gamma to LN. For clarity, we are using thresholding in this context to segment the vascular areas of the tumor and not to omit any regions from the analysis.


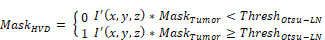

(9)

The complementary mask of LVD regions was obtained via subtraction of HVD from the tumor region of interest (ROI). The areas of the tumor considered to be avascular (AV) from the noise thresholding described in section 2.5 were not included in the LVD regions as shown in EQ. 10.


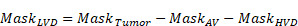

(10)

**Figure 2:**
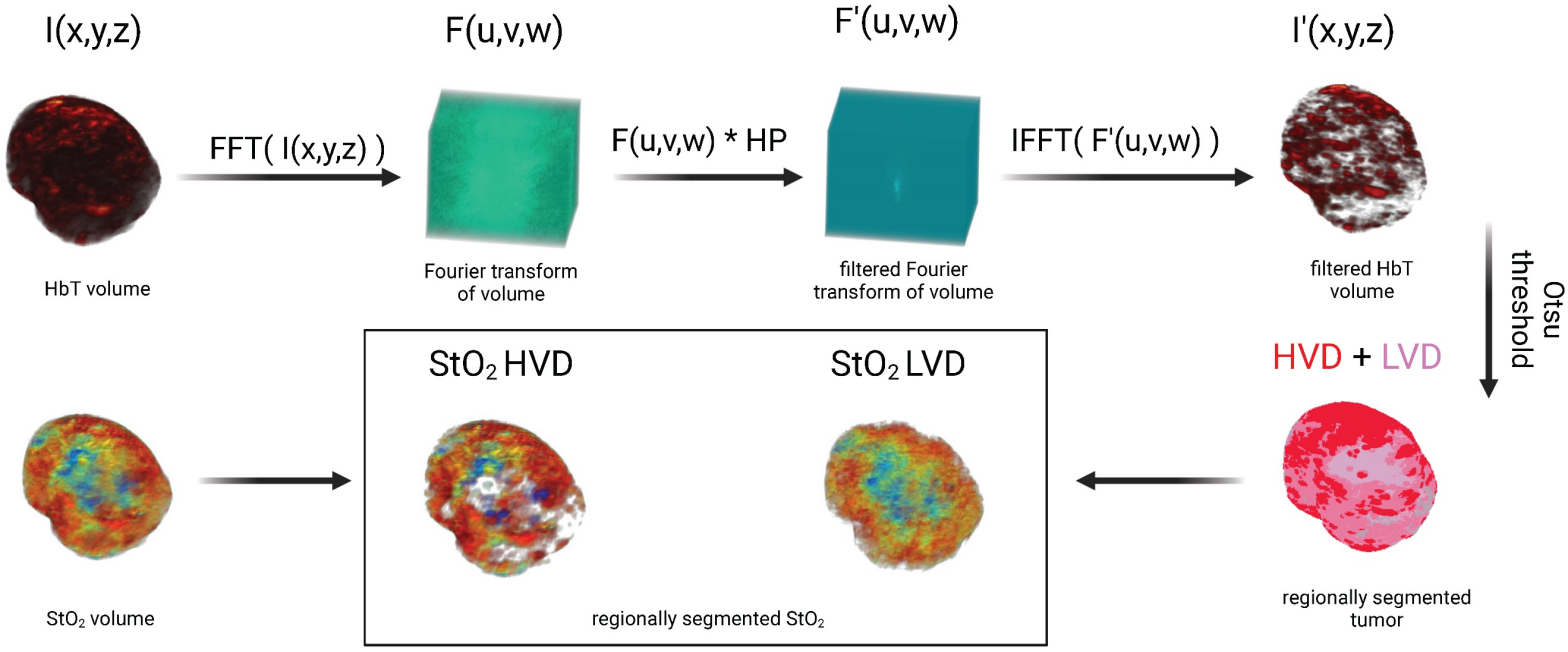
Image processing workflow displaying method for segmenting regions of high vascular density (HVD) and low vascular density (LVD)

This process allowed the regional analysis of the StO₂ maps as shown in Figure 2. As the majority of the low-frequency image components were removed with filtering, no analysis was performed on the filtered HbT images. Instead, the filtered images were solely utilized to define the vascular regions, which were then applied to the non-filtered HbT and StO₂ images.

### Immunohistochemistry

#### Sectioning and staining procedures

Post-euthanasia, tumors were surgically removed with the skin and then placed in optimal cutting temperature (OCT) compound (Tissue-Tek) in the same orientation as the B-scan US-PA images. The tumor tissue was carefully sliced into cryo-sections, each measuring 10 µm in thickness, using a cryotome, then securely affixed to glass microscope slides. The histological examination was conducted using the hematoxylin and eosin (H&E) staining method, as well as immunofluorescence (IF) staining, following a previously reported protocol 83. Briefly, the cryo-sections were fixed in ice-cold acetone and methanol solution (1:1 v/v) for 10 min and then air dried for 30 min, followed by three consecutive 5-minute washes with 1x phosphate-buffered saline (PBS). The tissue sections were then blocked with a 1x concentration blocking solution (Blocker™ BSA; #37525, ThermoFisher Scientific™) for 1 hour at room temperature. The immunostaining of vasculature within the tumor sections was performed using two primary antibodies, namely the Mouse PECAM-1/cluster of differentiation 31 (CD31) Affinity Purified Polyclonal Ab (#AF3628, R&D Systems Inc) and Rabbit ACTA2/alpha-Smooth Muscle Actin (aSMA) Polyclonal Ab (#50-556-90, Fisher Scientific). Tissue sections adjacent to sections chosen for CD31 and SMA were used to stain for the proliferation marker Ki-67 using the primary antibody Human Ki-67/MKI67 Antibody (#AF7617, R&D Systems Inc) The tissue sections were incubated overnight at 4 °C with the antibodies at dilutions of 1:5, 1:1000, and 1:20 for CD31, aSMA, and Ki-67, respectively. The primary antibodies were washed off with three rinses of 1x PBS the next day. Secondary antibody Donkey Anti-Goat IgG NL637 Affinity Purified PAb for CD31 (#NL002, R&D Systems Inc), Donkey Anti-Rabbit IgG (#NL004, R&D Systems Inc) for aSMA, and Donkey Anti-Sheep IgG (#NL010, R&D Systems Inc) for Ki-67, all at a dilution of 1:200, were added to the tissue sections and incubated for 2 h at room temperature. After the incubation, the sections were rinsed in PBS and the nuclei were counterstained and mounted with Slowfade gold antifade mountant containing 4',6-diamidino-2-phenylindole (DAPI; #S36939, Invitrogen). The slides were imaged at a 20X magnification using EVOS M7000 (ThermoFisher Scientific™) fluorescence imaging system. The IF-stained slides were imaged at the same brightness for all intensities using appropriate filter cubes. Tumors extracted on D(3) were stained with only CD31, while tumors extracted on D(8) were stained with CD31, aSMA, and Ki-67.

#### Immunofluorescence correlation with photoacoustic images for VRA validation

To validate the vascular segmentation algorithm, histological evaluation was performed using the endothelial marker CD31 with a complementary DAPI stain. The corresponding PA cross-section was determined using fiducial markers from the US images and matched to the closest H&E section as previously described 108. Prior to correlation, IF images were thresholded to a level where autofluorescence was negligible and the tumor region was segmented from the DAPI stain and applied to all IF images. The MATLAB 'regionprops' function was used to find the bounding box of the tumor region in the IF images and the complementary bounding box of the tumor region from US-PA images. After cropping all images to the size of their bounding box, the IF images were down sampled to the size of the US-PA images. Thirion's demons algorithm 109, 110 implemented via the MATLAB 'imregdemons' function was used to co-register the down-sampled IF tumor mask with the US-PA tumor mask and was visually confirmed. Once co-registered, 1 mm x 1 mm rectangular ROIs were drawn, covering the entire tumor region to correlate the average CD31 signal intensity with the fraction of HVD pixels in that same area. Average CD31 signal intensity was calculated as the sum of CD31 intensity in a region divided by the total number of pixels in the region. This parameter was directly correlated with the fraction of HVD pixels, calculated as the sum of pixels labeled as HVD divided by the total number of pixels in the region. For the correlation analysis, 1 cross-section was analyzed for 3 different tumors, giving 91 total ROIs, each containing relative amounts of LVD and HVD. To ensure that the segmentation method worked independently of tumor size, vascularity, and treatment regimen, the tumors analyzed all differed in volume (V = 83.9, 246.4, and 105.6 mm^3^), vascular parameters (StO₂ avg = 53.2%, 81.1%, 58.4%), and encompassed both treatment groups (sunitinib, vehicle, sunitinib).

#### Immunofluorescence to examine cell proliferation

We utilized the Ki-67 stain to measure the relative amounts of cell proliferation between both cell lines and treatment conditions at D(8). For both AsPC-1 and MIA PaCa-2, we analyzed 3 different tumors with a minimum of 5 total sections for each treatment group. Any sections with apparent image artifacts were omitted from the analysis. All images were normalized and thresholded to a level where autofluorescence was negligible and the DAPI and Ki-67 images were binarized for quantification. We defined the Ki-67 or proliferation index as the ratio of Ki-67 positive to DAPI positive pixels (Ki-67^+^ / DAPI^+^) over the entire tumor region, which was segmented from the DAPI image.

#### Immunofluorescence to determine the extent of vascular normalization

To confirm that the VRA measurements of HVD StO₂ and HbT were indicative of vascular remodeling, we resected 3 tumors from each treatment group on D(8) and conducted histological analysis. To this end, we performed triple sequential staining of endothelial cells, pericytes, and perfusing vessels using CD31, aSMA, and TL respectively with a DAPI counterstain. For AsPC-1 and MIA PaCa-2 sections from 3 different tumors were analyzed with a minimum of 5 total sections from each treatment group. Sections were omitted from quantitative analysis if there was the presence of significant image artifacts. Before analysis, IF images were normalized and thresholded to a level where autofluorescence was negligible and the CD31, TL, and aSMA images were binarized for quantification. The vascular normalization index (VNI) was calculated as the ratio of aSMA positive to CD31 positive pixels (aSMA^+^ / CD31^+^) for the entire tumor region in each section and averaged. Vascular perfusion was quantified in the same way, as the ratio of lectin-positive pixels to CD31-positive pixels (TL^+^ / CD31^+^). The tumor region was segmented from the DAPI stain and applied to all channels for each specific section.

### Statistical analysis

GraphPad Prism (La Jolla, CA) was utilized to execute all statistical analyses. Pearson correlation analysis (two-tailed) was performed to validate the vascular segmentation algorithm described above. The Pearson's correlation coefficient was calculated for each tumor individually and en masse. Pearson's correlation analysis was also performed between our histological and PA imaging metrics across all tumors quantified. Volume growth rates were calculated from the exponential fitting of the volume growth curves for each mouse. The fit growth rate value between groups was compared using the extra sum-of-squares F test. Growth rate values were omitted from the analysis in the case of poor fitting due to non-treatment effects (R^2^ < 0.6). For statistical comparison between two groups with equal variance at a specific time point, an unpaired two-sample t-test was performed. When comparing two groups of unequal variances, Welch's t-test was performed. To statistically compare HVD with LVD parameters, a one-tailed paired t-test was conducted as we were looking to see if there was a significant change in one direction. When comparing more than two unpaired groups (NT and sunitinib treated) and two or more different cell lines (MIA PaCa-2, BxPC-3, AsPC-1), ordinary two-way ANOVA was utilized (Fisher's LSD Test). A p-value < 0.05 was considered statistically significant for all analyses.

## Results

### Validation of vascular segmentation algorithm with immunohistology

**Figure 3:**
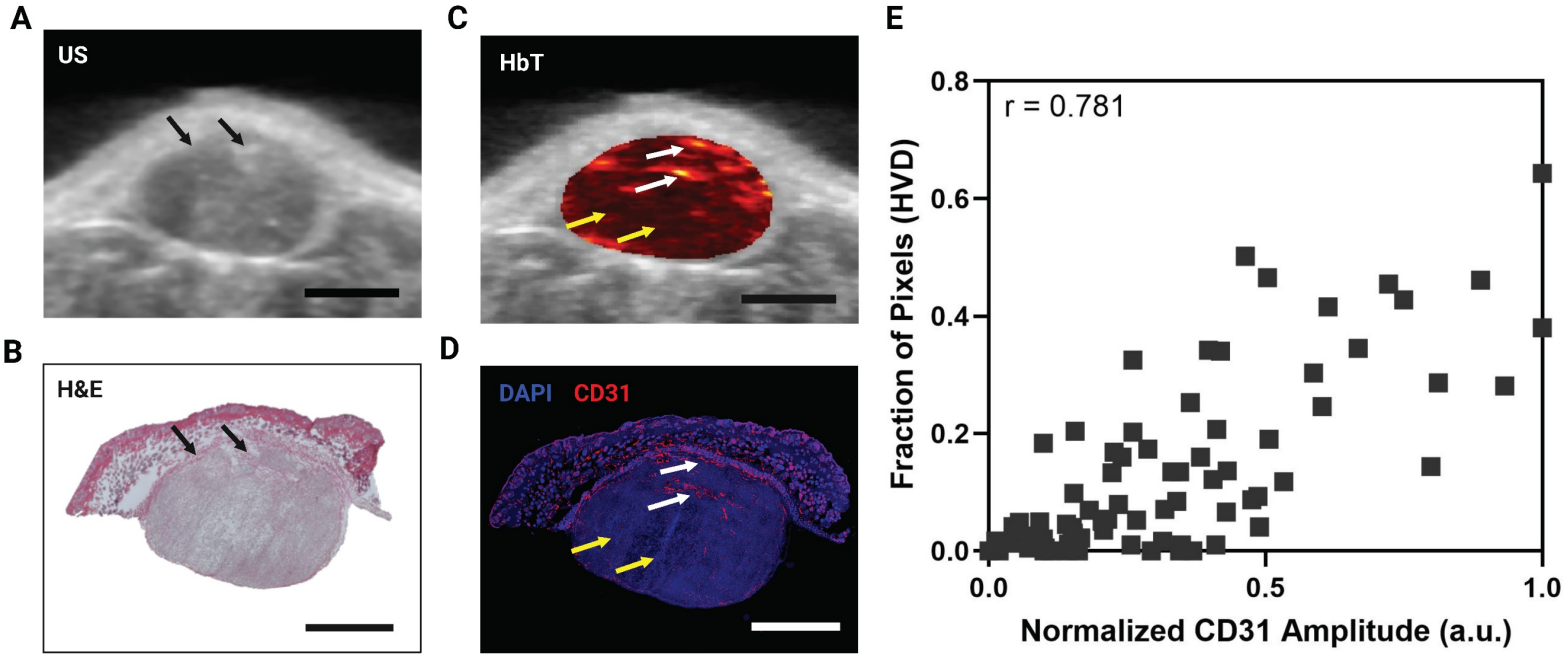
** A)** 2D cross sectional US image in a representative MIA PaCa-2 tumor. **B)** H&E stain of the same tumor cross section shown in A,C. **C)** 2D cross sectional image of HbT overlayed on US in a representative MIA PaCa-2 tumor with white arrows pointing towards areas of HVD and yellow arrows pointing towards areas of LVD. **D)** IF stain of the same tumor cross section shown in A,C with blue representing DAPI and red representing CD31. White arrows pointing towards areas of HVD and yellow arrows pointing towards areas of LVD according to the HbT image. **E)** Plot of normalized average CD31 intensity in a 1 mm x 1 mm ROI versus the fraction of pixels in the ROI labelled as HVD. All scale bars shown represent 2 mm.

Qualitatively there is a strong visual resemblance between the US image and the H&E stain of a representative tumor as shown by the tumor shape and fiducial markers (Figure 3A, B, black arrows). The HbT image of the same frame (overlayed onto the US image) and the corresponding CD31 stain show excellent visual correlation (Figure 3C,D). The areas labeled as HVD regions (white arrows) match areas of high CD31 signal, whereas the areas labeled as LVD regions (yellow arrows) match the areas of low CD31 signal intensity. The correlation between the average CD31 amplitude within a region and the fraction of pixels labeled HVD for 3 representative tumors is shown in Figure 3E. Each point on the plots shown represents a 1 mm x 1 mm ROI. The data points and ROIs that correspond to each tumor are separately plotted in Figure S1 and the data points for LVD + AV Fraction plotted against average CD31 amplitude is provided in Figure S2. Pearson's correlation coefficient indicates a strong correlation between CD31 and HVD for each mouse (r = 0.853, 0.704, 0.856) and en masse (r = 0.781). The p-values for Pearson's r are listed in Table S3. The strong quantitative and qualitative correlation between the pixels labeled HVD and CD31 signal intensity across several tumors' points to the reliability and repeatability of the proposed vascular segmentation algorithm.

### Effect of sunitinib on tumor growth in PC

**Figure 4:**
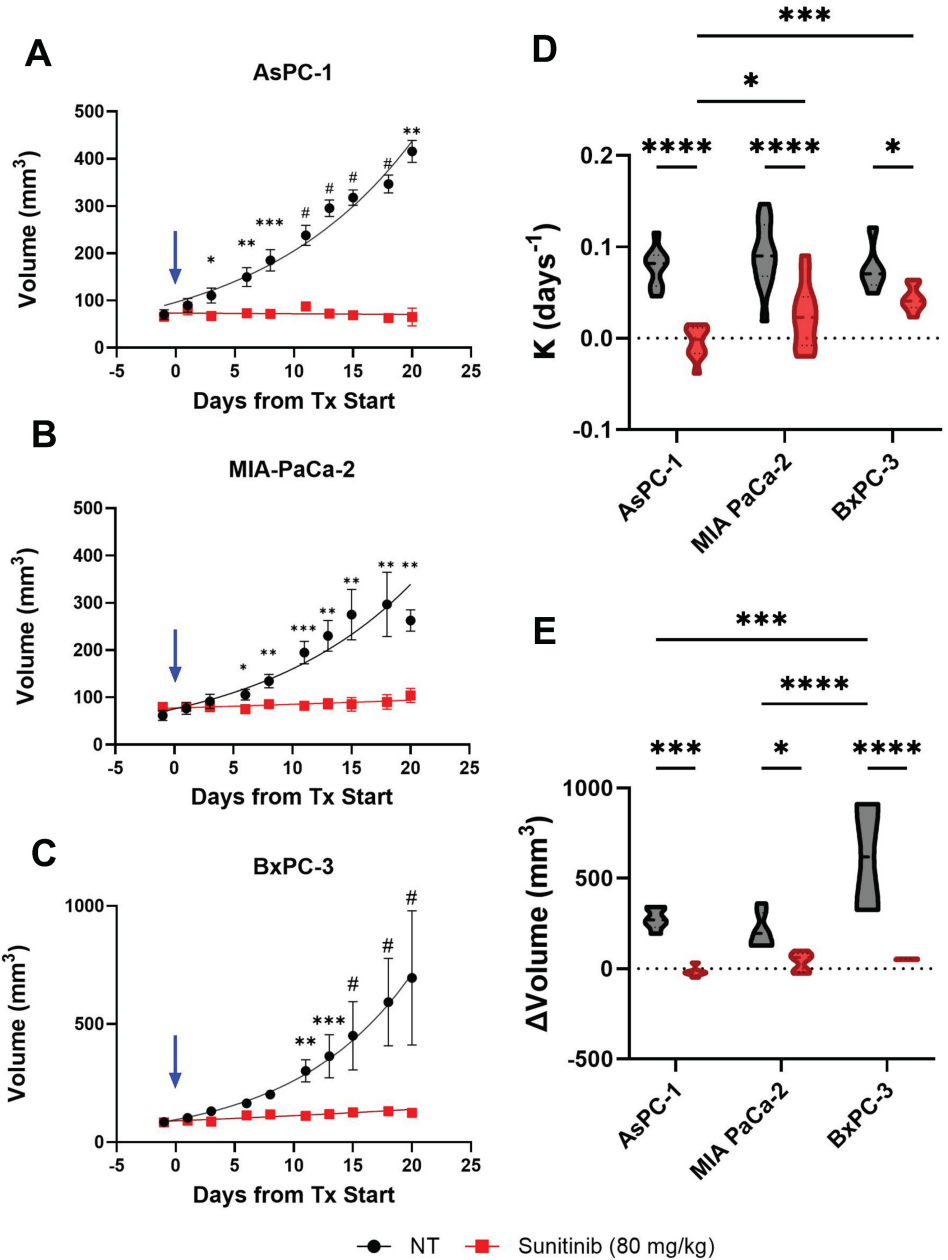
** A-C)** Plot of tumor volume for sunitinib (red) and no treatment - NT (black) groups for AsPC-1 (A), MIA PaCa-2 (B), and BxPC-3 (C) tumors with treatment starting at Day 0 (blue arrow). **D-E)** Violin plot of growth rate (D) and volume change from pre-treatment (D(-1)) to post-treatment or experiment end point (D(18-20)) (E) for sunitinib treated (red) and control tumors (black) in AsPC-1, MIA PaCa-2, and BxPC-3 tumors. All error bars shown represent SEM. p-values: * < 0.05, ** < 0.01, *** < 0.001, # or **** < 0.0001

Treatment with sunitinib greatly reduced tumor growth rate in both AsPC-1, MIA PaCa-2, and BxPC-3 xenografts as shown in Figure 4A, B and C respectively. A significant difference in tumor volume between the treated and control groups was apparent within 3, 6, and 11 days of treatment for AsPC-1, MIA PaCa-2, and BxPC-3 respectively. Applying an exponential fit to each treatment group reveals that for non-treated tumors, AsPC-1 (k = 0.0463) had a lower best-fit value for growth rate (k) than MIA PaCa-2 (k = 0.0905) and BxPC-3 (k = 0.1006) tumors, even though non-treated AsPC-1 tumors reached a larger volume than MIA PaCa-2 by D(20). Our findings were consistent with several studies utilizing subcutaneous pancreatic xenografts, in which quantitative measures of tumor volume showed that AsPC-1 tumors grew larger than MIA PaCa-2^111^, ^112^.

The difference between the average growth rate calculated for each cell line and treatment group is shown in Figure 4D and reveals that the average growth rates were significantly different between AsPC-1 and BxPC-3 treated tumors (p-value < 0.001) as well as AsPC-1 and MIA PaCa-2 treated tumors (p-value < 0.05). There was also a significant difference in the growth rate between sunitinib-treated and control tumors in AsPC-1 (p-value < 0.0001), MIA PaCa-2 (p-value < 0.0001), and BxPC-3 (p-value < 0.05) groups respectively. The growth rates of the non-treated tumors were insignificant between all cell lines (p-value > 0.05). The observed tumor volume changes throughout the treatment period are shown in Figure 4E. No significant difference was observed when comparing the three cell lines for the treated groups. Although the non-treated BxPC-3 tumors reached a significantly higher volume than both AsPC-1 (∆x̄ = 343.6 mm^3^, p-value < 0.001) and MIA PaCa-2 (∆x̄ = 409.8 mm^3^, p-value < 0.0001) tumors. Within the three different tumor types, the volume change was significantly higher for the control group compared to treated groups for AsPC-1 (x̄ = 292.7 mm^3^, p-value < 0.001), MIA PaCa-2 (∆x̄ = 170.3 mm^3^, p-value < 0.05), and BxPC-3 (∆x̄ = 566.4 mm^3^, p-value < 0.0001). Descriptive statistics reveal that treated AsPC-1 tumor volume was reduced by an average of 17.46 mm^3^ (σ = 27.11 mm^3^) and that tumor volume reduction due to sunitinib was consistent between mice. Alternatively, treated MIA PaCa-2 and BxPC-3 tumors showed an average volume increase of 38.69 mm^3^ (σ = 53.71 mm^3^) and 52.41 mm^3^ (σ = 3.67 mm^3^) during the sunitinib regimen with MIA PaCa-2 displaying a much larger variation in response.

**Figure 5:**
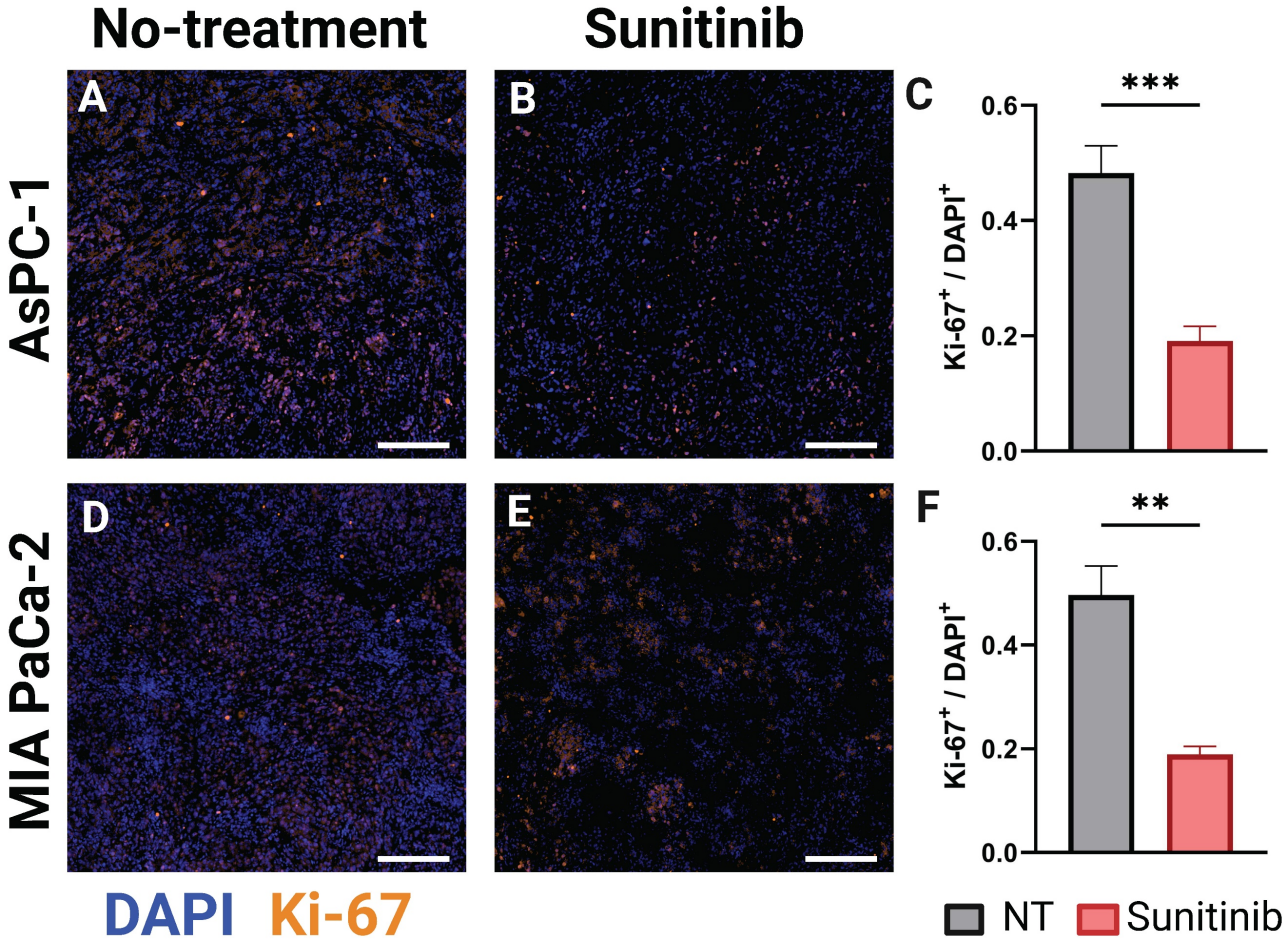
** A-B)** Representative AsPC-1 tumors stained for Ki-67 (orange) overlaid on DAPI (blue) for the control (NT) (A) and sunitinib treated (B) group.** C)** Bar graph comparing Ki-67^+^ / DAPI^+^ cell ratio between the control (black) and sunitinib (red) groups. **D-E)** Representative MIA PaCa-2 tumors stained for Ki-67 (orange) overlaid on DAPI (blue) for the control (D) and sunitinib treated (E) group.** F)** Bar graph comparing Ki-67^+^ / DAPI^+^ cell ratio between the control (black) and sunitinib (red) groups for MIA PaCa-2. All scale bars = 125 μm, all error bars shown represent SEM. p-values: * < 0.05, ** < 0.01, *** < 0.001

Ki-67 is an established marker for cellular proliferation 113. In an analysis of more than 500 resected clinical PC samples, Ki-67 was identified as an independent prognostic marker for overall survival and recurrence-free survival. Patients exhibiting low expression (Ki-67 index ≤ 30% or 0.3) demonstrated significantly greater overall survival compared to those with high expression levels (Ki-67 index > 30% or 0.3), irrespective of cellular differentiation 114. Prior data, concentrating on PC, also found that elevated Ki-67 expression correlated with adverse pathological characteristics, such as poor tumor differentiation, high tumor grade, and the development of lymph node metastases 115, 116, and was an independent predictor of unfavorable disease-free survival and disease-specific survival outcomes in PC and had a strong correlation with tumor grade 116-118. Leveraging the results of these studies, we examined the differences in Ki-67 expression in untreated and sunitinib treated AsPC-1 and MIA PaCa-2 tumors. In Figure 5, the stain shown in orange overlaid represents Ki-67, while the nuclear stain DAPI is shown in blue, for representative AsPC-1 (Figure 5A-B) and MIA PaCa-2 (Figure 5D-E) tumors. The Ki-67 index (Ki-67^+^ / DAPI^+^) was significantly higher in the non-treated tumors in both AsPC-1 (∆x̄ = 0.29, p-value < 0.001) and MIA PaCa-2 (∆x̄ = 0.31, p-value < 0.01) as shown in the bar graphs in Figure 5C and Figure 5F respectively. Of note, the average proliferation index of treated AsPC-1 (x̄ = 0.19) and MIA PaCa-2 (x̄ = 0.19) tumors fall into the low expression category from a clinical standpoint, while the non-treated groups fall in the high expression category. This data is corroborated with the findings of Liang *et al.* 114.

### Regional vascular response of pancreatic xenografts to sunitinib

#### AsPC-1 tumors

**Figure 6:**
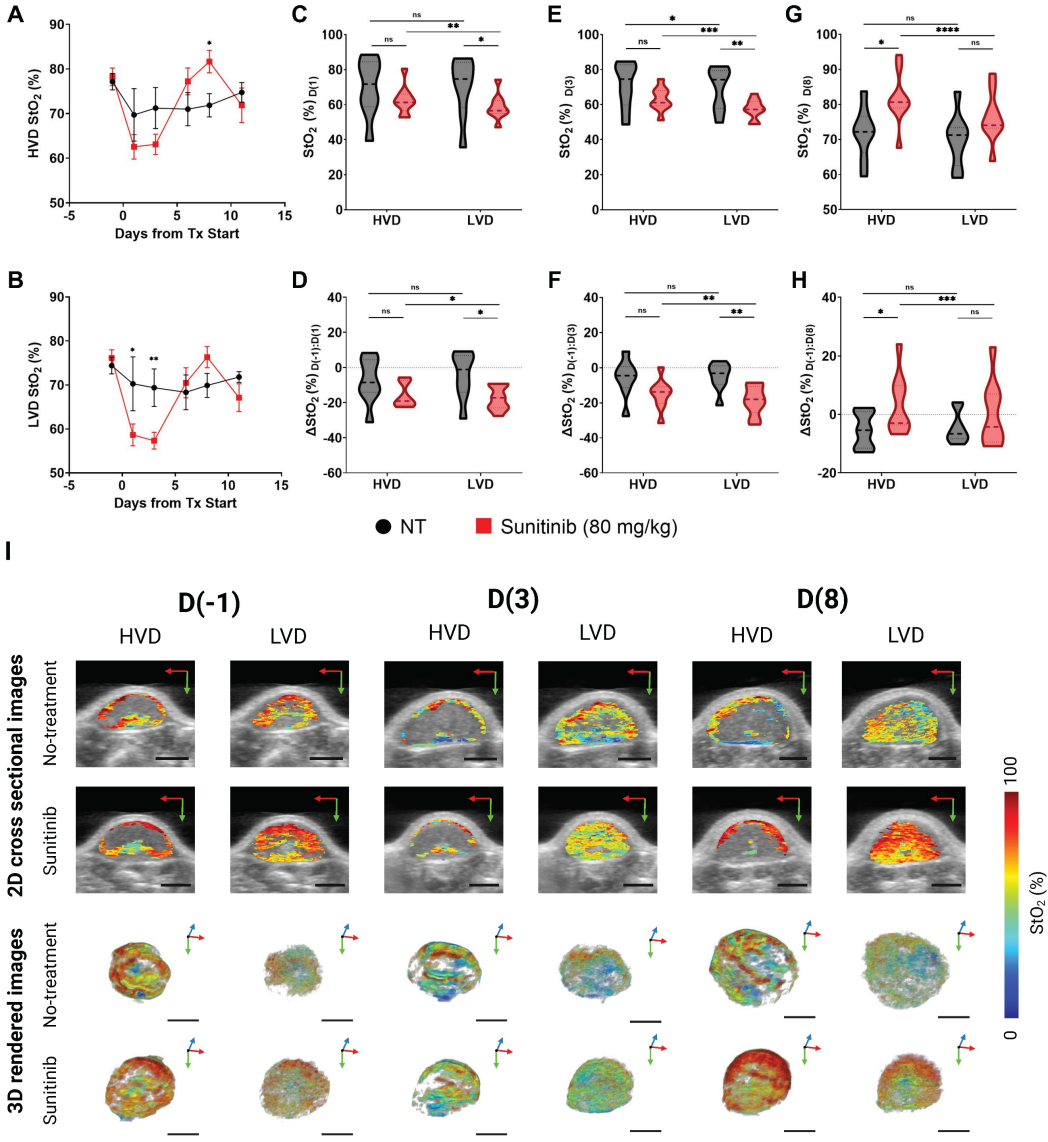
**A-B)** Plot of StO₂ in sunitinib (80 mg/kg) treated (red line) and untreated control (no treatment, black line) AsPC-1 tumors in regions of high vascular density (HVD) regions (A) and (LVD) regions (B). **C-D)** Violin plot comparing StO₂ on D(1) (C) and ∆StO₂ between D(1) and D(-1) (D) for Sunitinib at 80 mg/kg (red) and No Treatment (black) AsPC-1 tumors in areas HVD and LVD.** E-F)** Violin plot comparing StO₂ on D(3) (E) and ∆StO₂ between D(3) and D(-1) (F) for Sunitinib at 80 mg/kg (red) and No Treatment (black) AsPC-1 tumors in areas HVD and LVD. **G-H)** Violin plot comparing StO₂ on D(8) (G) and ∆StO₂ between D(8) and D(-1) (H) for Sunitinib at 80 mg/kg (red) and No Treatment (black) AsPC-1 tumors in areas HVD and LVD. **I)** Regional 2D cross sectional images (top) and 3D rendered images (bottom) of StO₂ in sunitinib (80 mg/kg) and no treatment AsPC-1 tumors displaying high HVD and LVD areas. All error bars shown represent SEM. Scale bars = 2 mm. p-values: * < 0.05, ** < 0.01, *** < 0.001, # or **** < 0.0001

The treatment-induced StO₂ changes in the AsPC-1 tumors are displayed quantitatively and qualitatively in Figure 6A-H and Fig 6I respectively. Representative 2D cross sectional image (typically from the center of the tumor) and 3D maps of tumor StO_2_ are shown for the same tumor across various time points on the top and bottom panel of Figure 6I respectively. The corresponding HbT data and images for the StO₂ images shown in Figure 6I are provided in Figure S3. The images depict ultrasound imaging in grayscale overlaid with pseudo colormap where blue is low oxygenated areas and red is highly oxygenated regions. When comparing average 3D StO₂ of the entire tumor volume in treated versus non-treated groups (Figure S4), the sunitinib group has lower StO_2_ at 24 h or D(1) (∆x̄ = -10.29%, p-value < 0.05) and at 72 h or D(3) post-treatment (∆x̄ = -9.80%, p-value < 0.05). These findings align with previous work from our group that has shown these time points to be significant for StO₂ reduction in PC xenografts treated with the VEGF inhibitor cabozantinib 82. Segmenting the tumor regions into areas of HVD and LVD revealed that sunitinib preferentially induced these StO₂ changes in LVD areas (Figure 6B) early in the treatment regimen. As seen in Figure 6A-C there was no significant difference in StO₂ or ∆StO_2_ between the treated and non-treated tumors at 24 h post-treatment (D(1)) in HVD areas (Figure 6C,D) . The 2D and 3D images at this time point shown in Figure 6I also depict similar HVD profiles. Alternatively, there was a strong significant difference between the StO₂ values of the two treatment groups in LVD areas at the same time-point (∆x̄ = -11.61%, p-value < 0.05). A significant difference in StO₂ between the HVD and LVD areas was also seen at 24 h post-treatment in the sunitinib-treated group (p-value < 0.001), whereas no s difference was seen between the vascular regions for the control tumors (Figure 6C, grey bars).

At the 72-hour post-treatment time point D(3), significant differences in StO₂ remain between the treated and control group for LVD (∆x̄ = -12.05%, p-value < 0.01) with HVD regions still showing no significant difference (Figure 6E). Substantial changes in oxygen saturation (∆StO₂) from pre-treatment values (D(-1)) were seen at time points D(1), D(3), and D(8) respectively (Figs. 6D,F,H). The ∆StO₂ in LVD regions was significantly different between treated to control tumors on D(1) (∆x̄ = -13.33%, p-value < 0.05) and D(3) (∆x̄ = -13.77%, p-value < 0.01). No significant differences between the treatment groups were observed in HVD regions on D(1) or D(3). The HVD images in 6I also depict the same where StO_2_ in the HVD regions remained relatively high in both treated and untreated groups at all time points. Additionally, significant differences were observed between the LVD and HVD regions only in the sunitinib group within the first 24 h (∆x̄ = -1.8%, p-value < 0.05) and 72 h (∆x̄ = -3.66%, p-value < 0.01) of the regimen. Upon visual inspection of Figure 6I, it can be observed that 1. the HVD regions are mainly localized to the tumor periphery in both the treated and non-treated AsPC-1 tumors and 2. Sunitinib treatment caused significant StO_2_ changes mostly in the LVD regions. Given these results, we believe that sunitinib is preferentially targeting LVD areas within the first 72 h of the treatment regimen in AsPC-1 xenografts.

The key time points associated with treatment-induced StO₂ decrease were observed within the first 72 h of the treatment regimen, however, we also examined the longer-term effect of sunitinib on tumor vasculature. Specifically, drastic reoxygenation of the HVD regions was observed by D(6) in half of the treated tumors and by D(8) in all but one treated tumor. The reoxygenation is visually apparent in Figure 6I images, particularly in the HVD images that at D(8) sunitinib treated tumors have reoxygenated. The amount of reoxygenation that occurred between D(-1) and D(8) was significant in HVD compared to LVD regions (∆x̄ = 3.19% , p-value < 0.001). The HVD regions in treated tumors showed increased D(8) StO₂ (∆x̄ = 9.78%, p-value < 0.05) and ∆StO₂ (∆x̄ = 8.70%, p-value < 0.05) when compared to the control group (Figure 6G,H). Additionally, from D(3:8) the oxygenation status of HVD regions increased by >20% on average in treated tumors, which can be clearly seen in the 2D and 3D images shown in Figure 6I. On the other hand, there was a minimal change in tumor StO_2_ in the no-treatment group, despite the increase in tumor volume during these timepoints as displayed by the 3D renders. No significant difference was seen in StO₂ and ∆StO₂ for the LVD or HVD regions of control tumors at D(8), indicating that the reoxygenation is related to sunitinib-induced vascular changes.

While StO₂ and ∆StO₂ are both excellent indicators of vascular changes within the TME 82-84, 119, we also examined the regional changes in HbT to determine if the observed increase in StO₂ could be attributed to vascular remodeling or cyclic changes in tumor oxygenation. The regional HbT changes throughout the D(-1:11) period are shown in Figure S3, while the whole tumor HbT changes are quantified in Figure S4. Sunitinib-treated tumors had significantly higher HVD HbT signal at D(6) (∆x̄ = 6.43e-4 a.u., p-value < 0.05) and D(8) (∆x̄ = 6.35e-4 a.u., p-value < 0.05) compared to the vehicle group. No significant differences between the treatment groups were seen for HbT in LVD regions at these time points. The change in HbT (∆HbT) from D(-1:8) was also significantly different between the treated and control tumors in HVD regions (∆x̄ = 8.17e-4 a.u., p-value < 0.001), and showed that the average HVD HbT increased from pre-treatment levels in the treated group, while decreasing in the control group. Given the insignificant changes in hemoglobin content in LVD regions, and increase in HVD HbT and StO₂, the increased blood content in HVD regions is indicative of improved blood flow and vessel functionality at this juncture.

These observations provide strong evidence that vascular normalization is occurring within the D(4:8) window. From our observations, it seems highly likely that sunitinib initially led to a temporary decrease in tumor StO₂ while vessels were ablated prior to the onset of vascular remodeling. We see evidence of the preferential ablation of vessels in the LVD regions, indicating these regions housed more immature vessels. Once these immature vessels are pruned, a subsequent increase in HVD StO₂ and HbT is observed, providing surrogate markers for tissue reoxygenation and improved blood flow. These structural and functional changes occur more prominently in the HVD regions indicating the remodeling of more mature vessels in response to the preferential pruning of vessels in the LVD region. All of these observed changes are consistent with previous reports of vascular normalization 17, 23, 24, 51, 120, which we go on to histologically validate in further sections.

#### MIA PaCa-2 tumors

**Figure 7:**
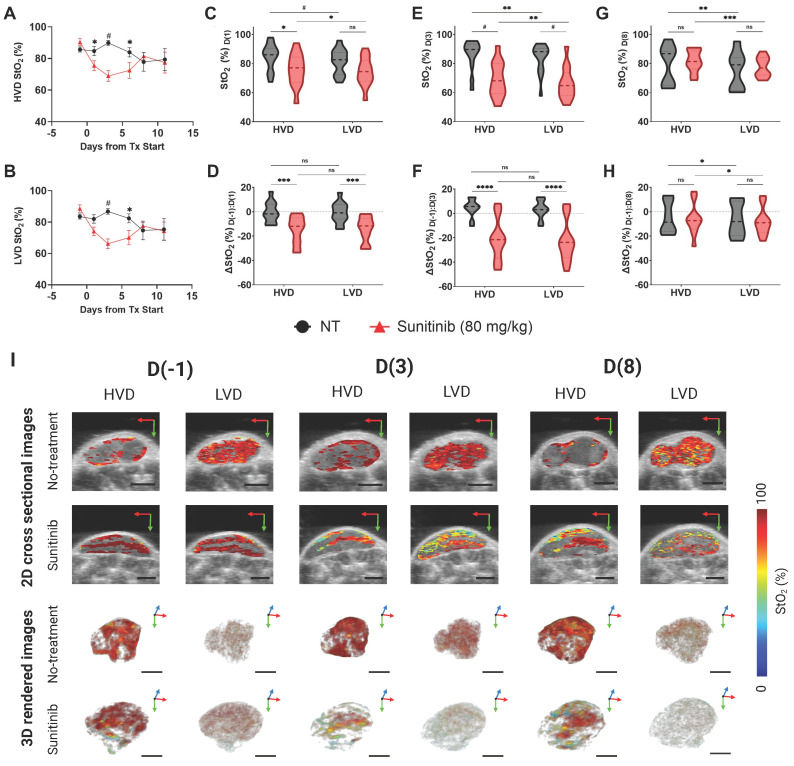
**A-B)** Plot of StO₂ in sunitinib (80 mg/kg) treated (red line) and untreated control (no treatment, black line) MIA PaCa-2 tumors in regions of high vascular density (HVD) regions (A) and (LVD) regions (B). **C-D)** Violin plot comparing StO₂ on D(1) (C) and ∆StO₂ between D(1) and D(-1) (D) for Sunitinib at 80 mg/kg (red) and No Treatment (black) MIA PaCa-2 tumors in areas HVD and LVD.** E-F)** Violin plot comparing StO₂ on D(3) (E) and ∆StO₂ between D(3) and D(-1) (F) for Sunitinib at 80 mg/kg (red) and No Treatment (black) MIA PaCa-2 tumors in areas HVD and LVD. **G-H)** Violin plot comparing StO₂ on D(8) (G) and ∆StO₂ between D(8) and D(-1) (H) for Sunitinib at 80 mg/kg (red) and No Treatment (black) MIA PaCa-2 tumors in areas HVD and LVD. **I)** Regional 2D cross sectional images (top) and 3D rendered images (bottom) of StO₂ in sunitinib (80 mg/kg) and no treatment MIA PaCa-2 tumors displaying high HVD and LVD areas. All error bars shown represent SEM. Scale bars = 2 mm. p-values: * < 0.05, ** < 0.01, *** < 0.001, # or **** < 0.0001

Next, we investigated the MIA PaCa-2 xenografts to determine if the preferential treatment of LVD regions by sunitinib in PC was cell-line dependent. The treatment-induced regional StO₂ changes in the MIA PaCa-2 tumors are quantitatively and quantitively displayed in Figure 7A-H, and Figure 7I respectively. The respective HbT images for the StO₂ images shown in Figure 7 are displayed in Figure S4. The average StO₂ of the whole tumor in treated and non-treated groups is significantly different at 72- h or D(3) post-treatment (∆x̄ = -21.04%, p-value < 0.0001) in the MIA PaCa-2 tumors (Figure S5). Compared to AsPC-1, the initial drop in StO₂ is over 10% greater in the MIA PaCa-2 tumors.

Interestingly, performing regional analysis on these tumors reveals a distinct trend from AsPC-1, i.e., in the MIA PaCa-2 tumors, sunitinib is inducing StO₂ changes non-preferentially during early treatment time points. There was a no significant difference in StO₂ between the treated and control tumors at 24 h post-treatment (D(1)) in LVD areas, while there was a statistical difference in the HVD areas (∆x̄ = -9.17%, p-value < 0.05) as shown in Figure 7A-C. However, this is not the case for the D(-1:1) ∆StO₂ in either region. By D(3) and D(6), there are significant differences between the StO₂ in the treated and control tumors in both LVD and HVD areas, indicating no significant regional trend was occurring. Reoxygenation also happened in the MIA PaCa-2 tumors after the early treatment period, however, this reoxygenation was more subtle. From D(3:8) we do observe reoxygenation in both the LVD and HVD regions of treated tumors, however, neither region reaches a StO₂ value above the pre-treatment baseline (Figure 7D,F,H), or a value greater than the control tumors at the same juncture (Figure 7C,E,G). Although the non-treated MIA PaCa-2 tumors displayed differing StO₂ levels in the HVD and LVD regions, the changes in these regions over time were consistent. Also, HbT decreased in the HVD and LVD areas for all treated mice during this window, unlike in AsPC-1. Regional HbT values from the D(-1:11) treatment days are provided in Figure S5 and show no significant differences in HbT or ∆HbT between the sunitinib and control group at these time points. The whole tumor StO_2_ and HbT values for the entire treatment duration can be found in Figure S4.

Several distinct differences between the AsPC-1 and MIA PaCa-2 tumors can be seen in the 2D and 3D images of StO_2_ in Figure 6I and 7I. Firstly, the untreated MIA PaCa-2 tumors remained highly oxygenated at D(8), unlike AsPC-1. Despite the MIA PaCa-2 tumors having a larger decrease in StO_2_ between D(-1) and D(3), the overall LVD StO_2_ in the MIA PaCa-2 treated tumors remained substantially higher (~10%) than that of AsPC-1 treated tumors on D(3). Neither average HbT nor StO₂ in sunitinib-treated mice was significantly higher than the controls and these values did not increase beyond pre-treatment levels indicating no improvement in vascular function. For these reasons, it is unlikely that any vascular normalization occurred during this window in the sunitinib-treated MIA PaCa-2 tumors, which is further confirmed by histological analysis. The increase in StO₂ was relatively small and was only observed for a short period, leading us to believe it is more likely due to cyclic fluctuations in tumor StO₂. Towards understanding these effects, our future work will involve monitoring the tumors at more frequent time points during the treatment regimen to investigate the prevalence of short-term fluctuations in StO₂.

#### BxPC-3 tumors

The AsPC-1 and MIA PaCa-2 have relatively low pro-angiogenic potential for PC cell lines, prompting us to examine the effects of sunitinib on the BxPC-3 cell line, which has significantly higher expression of pro-angiogenic factors 121. While the overall tumor StO₂ significantly decreased from D(-1:3) in both treated MIA PaCa-2 and AsPC-1, the treated BxPC-3 group did not display this initial drop in StO₂. Despite the LVD regions having significantly lower StO₂ and ∆StO₂ than HVD regions at D(1,3,8) in the treated group, none of the LVD or HVD StO₂ metrics were significantly different between treatment groups (Figure S6). From this we can garner that, while sunitinib impacted the oxygenation differently in LVD regions compared to HVD regions, these changes were not drastic enough for the StO₂ in either region to significantly deviate from the control. Additionally, ∆HbT (Figure S7) was not significant between treatment groups (sunitinib and control) or vascular regions (HVD and LVD). These results align with another study where BxPC-3 tumors treated with sunitinib did not have an increased hypoxia fraction compared to control mice 49. Furthermore, high vascular variability was present in the non-treated BxPC-3 mice prior to treatment onset (StO₂ s = 13.43%) compared to AsPC-1 (StO₂ s = 5.22%) and MIA PaCa-2 (StO₂ s = 7.46%). Alternatively, due to the higher pro-angiogenic potential of BxPC-3, a higher dose of sunitinib may be required to induce the preferential reduction of StO₂ in LVD regions at the time points we have investigated. This is further confirmed by the high IC-50 value of BxPC-3 cells treated with sunitinib *in vitro* reported by Liang *et al.* 122.

### Histological evaluation confirms sunitinib-induced vascular normalization in AsPC-1

**Figure 8:**
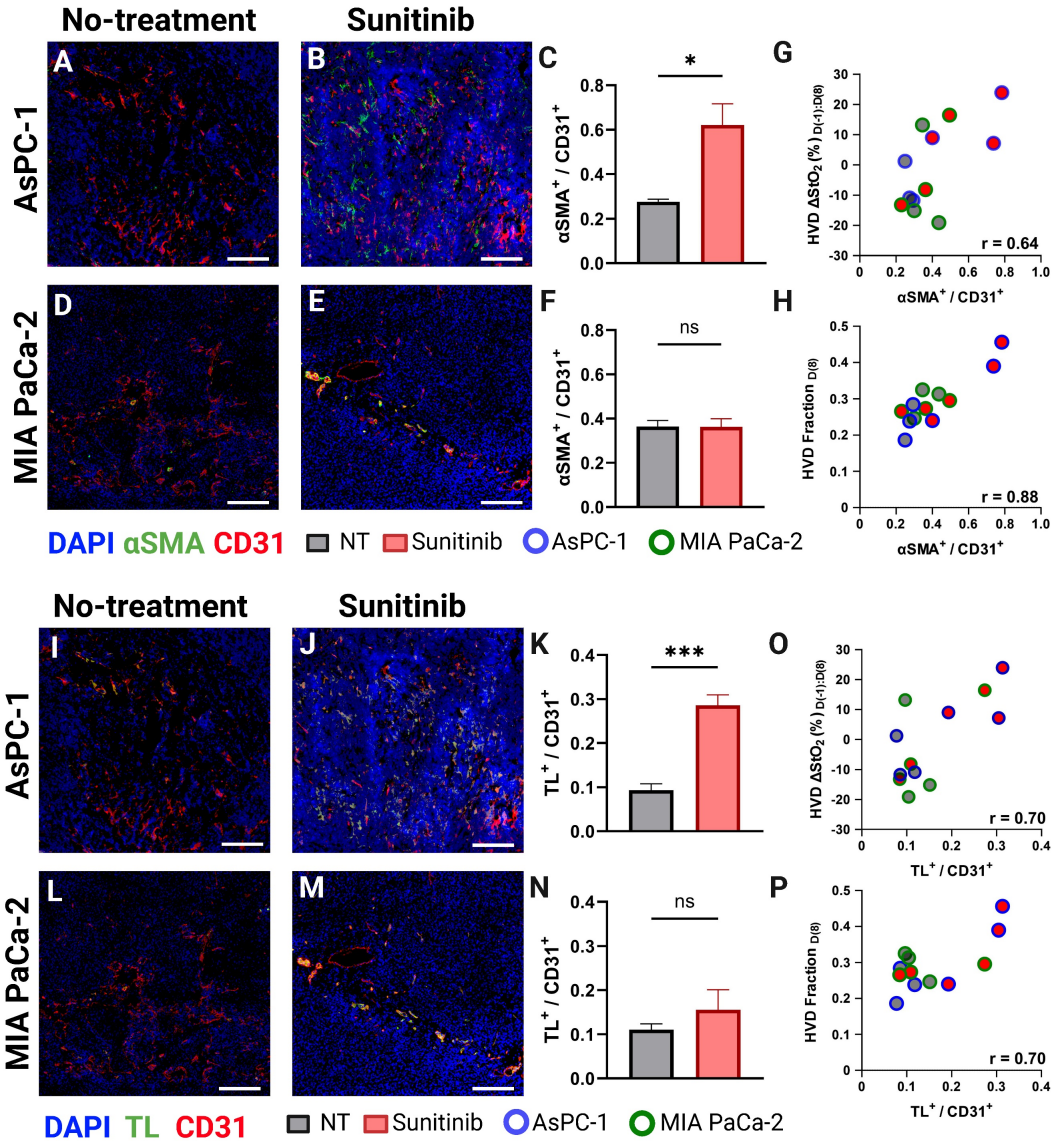
**A-B)** Representative AsPC-1 tumors stained for CD31 (red) and αSMA (green) overlaid on DAPI (blue) for the control (A) and sunitinib (B) treated group.** C)** Bar graph comparing αSMA ^+^ / CD31^+^ cell ratio between the control (black) and sunitinib (red) groups. **D-E)** Representative MIA PaCa-2 tumors stained with CD31 (red) and αSMA (green) overlaid on DAPI (blue) for the control (D) and sunitinib (E) treated group.** F)** Bar graph (Mean +/- SEM) comparing αSMA ^+^ / CD31^+^ cell ratio between the control (black) and sunitinib (red) groups for MIA PaCa-2. **G-H)** Scatter plot of αSMA^+^ / CD31^+^ versus the HVD ∆StO₂ between D(8) and D(-1) (G) HVD fraction on D(8) (H) for each histological sample with points corresponding to MIA PaCa-2 tumors outlined in green, and AsPC-1 tumors outlined in blue. **I-J)** Representative AsPC-1 tumors stained for CD31 (red) and TL (green) overlaid on DAPI (blue) for the control (I) and sunitinib (J) treated group.** K)** Bar graph comparing TL ^+^ / CD31^+^ cell ratio between the control (black) and sunitinib (red) groups. L**-M)** Representative MIA PaCa-2 tumors stained with CD31 (red) and TL (green) overlaid on DAPI (blue) for the control (L) and sunitinib (M) treated group.** N)** Bar graph comparing TL ^+^ / CD31^+^ cell ratio between the control (black) and sunitinib (red) groups for MIA PaCa-2. **O-P)** Scatter plot of ΤL^+^ / CD31^+^ versus the HVD ∆StO₂ between D(8) and D(-1) (O) HVD fraction on D(8) (P) for each histological sample with points corresponding to MIA PaCa-2 tumors outlined in green, and AsPC-1 tumors outlined in blue. It is to be noted that all four stains DAPI, TL, αSMA and CD31 are performed on the same section. For display purposes we showed DAPI, TL, CD 31 and DAPI, αSMA and CD31 images separately. All scale bars = 125 μm, All error bars shown represent SEM. p-values: * < 0.05, ** < 0.01, *** < 0.001

To confirm if VRA could provide surrogate markers of vascular normalization successfully, we conducted histological staining of CD31, αSMA, and TL on the frozen tissue sections. In mature blood vessels, the coordination between pericyte coverage, as evidenced by αSMA staining, and endothelial integrity, as indicated by CD31 expression, improves vascular stability and functionality 52, 70. Enhanced vessel permeability facilitates increased vascular perfusion by enabling a greater flux of plasma and its constituents, including nutrients and oxygen, into the surrounding tissue. This phenomenon can be quantitatively assessed using TL staining, a method that selectively binds to the glycoproteins on the luminal surface of endothelial cells, allowing for the visualization and measurement of vascular density and perfusion 123. Figure 8 displays representative AsPC-1 (Figure 8A-B) and MIA PaCa-2 (Figure 8D-E) tumors with CD31 (red), αSMA (green), and DAPI (blue). The TL stain (green) is shown for the same tissue cross-sections are shown in Figure 8G,H and J,K for AsPC-1 and MIA PaCa-2 respectively. Supporting our PA data, the VNI (αSMA^ +^ / CD31^+^) was significantly higher in the treated tumors for only the AsPC-1 (∆x̄ = 0.34, p-value < 0.05) cell line (Figure 8C and Figure 8F respectively). The observation of increased vessel functionality is further reinforced by the increased TL^+^ / CD31^+^ ratio shown in the treated AsPC-1 vasculature compared to the control (∆x̄ = 0.19, p-value < 0.001). The MIA PaCa-2 tumors treated with sunitinib did display an increase in the average TL^+^ / CD31^+^ ratio, however this increase was statistically insignificant.

We also correlated both αSMA^ +^ / CD31^+^ and TL^+^ / CD31^+^ with two of our VRA PA metrics (D(-1:8) HVD ∆StO₂ or D(8) HVD fraction (Figure 8G-H, O-P)) to investigate their relationship to vessel maturity and perfusion. The αSMA^ +^ / CD31^+^ metric had good correlation with D(-1:8) HVD ∆StO₂ (r = 0.642, p-value < 0.05) and excellent correlation with D(8) HVD fraction (r = 0.884, p-value < 0.001) with treated AsPC-1 tumors showing higher average of D(-1:8) HVD ∆StO₂ and HVD fraction compared to both treated MIA PaCa-2 tumors and all untreated tumors. The TL^+^ / CD31^+^ metric showed a good correlation with D(-1:8) HVD ∆StO₂ (r = 0.703, p-value < 0.05) and HVD fraction (r = 0.695, p-value < 0.05). Significant increases in HVD StO₂ and HVD fraction in a tumor undergoing anti-angiogenic therapy could serve as prognostic markers of vascular normalization or provide insights into vessel maturity and functionality* in vivo.*

### Vascular differences between pancreatic cancer cell lines

**Figure 9:**
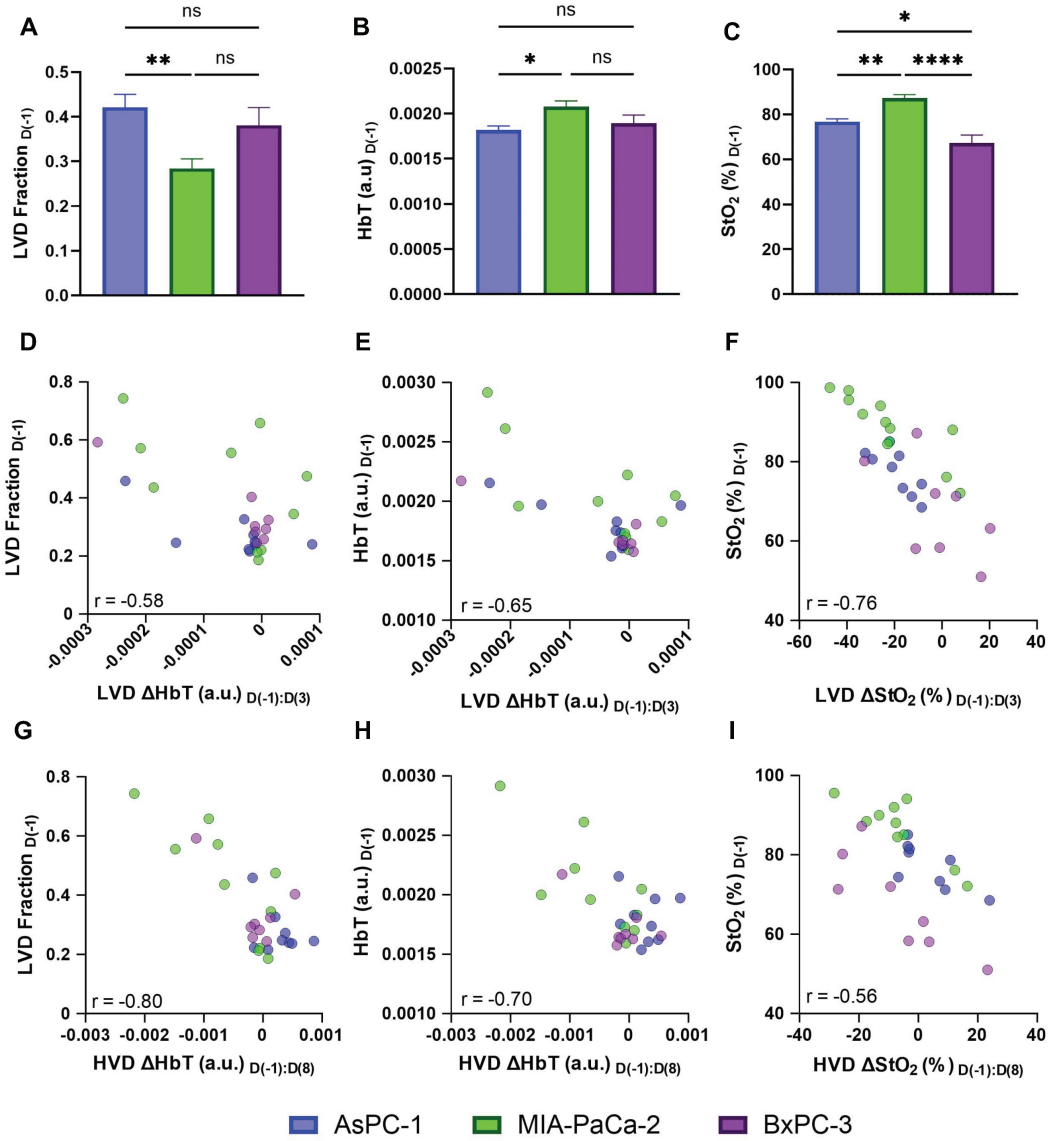
**A-C)** Bar graphs (Mean +/- SEM) comparing the pre-treatment LVD fraction **(A)**, HbT **(B)**, and StO₂ **(C)** in all AsPC-1 (blue) and MIA PaCa-2 (green) tumors and BxPC-3 (purple). **D-E)** scatter plots of LVD ΔHbT on D(3) versus pre-treatment LVD fraction (D), and HbT (E).** F)** scatter plot of LVD ΔStO_2_ from D(-1) to D(3) in sunitinib-treated tumors. ). G**-H)** scatter plots of HVD ΔHbT on D(8) versus pre-treatment LVD fraction (D), and HbT (E).** I)** scatter plot of HVD ΔStO_2_ from D(-1) to D(8) in sunitinib-treated AsPC-1 (blue), MIA PaCa-2 (green), and BxPC-3 (purple) tumors. p-values: * < 0.05, ** < 0.01, *** < 0.001, **** < 0.0001

While the effect of sunitinib on tumor volume between the AsPC-1, MIA PaCa-2, and BxPC-3 cell lines was comparable (Figure 4), the regional effect on the vasculature of the three cell lines was markedly different. To understand the discrepancy in response seen, we investigated the baseline vascular characteristics of the tumors from these cell lines. As shown in Figure 9A there was a difference in the pre-treatment LVD Fraction of AsPC-1 and MIA-PaCa-2 (p-value < 0.01) with AsPC-1 tumors displaying a significantly higher fraction of LVD areas. When looking at the whole tumor volume, the pre-treatment HbT values between AsPC-1 and MIA PaCa-2 were also significantly different on D(-1) (Figure 9B, p-value < 0.05), while the pre-treatment StO_2_ was different between all cell lines. Figure 9D displays that the StO₂ was higher in MIA PaCa-2 tumors than in BxPC-3 (∆x̄ = 12.88%, p-value < 0.0001) and AsPC-1 (∆x̄ = 17.16%, p-value < 0.01) with BxPC-3 StO₂ being slightly lower than AsPC-1 (∆x̄ = -1.89%, p-value < 0.05). The pre-treatment StO₂ and HbT as well as the relative amounts of LVD regions in a tumor may pre-dispose it to vascular remodeling with sunitinib, leading us to investigate the relationship between these parameters and the VRA metrics displaying key vascular features. We correlated our pre-treatment parameters with our VRA metrics for vascular pruning from D(-1) to D(3) in LVD regions and improved vessel function from D(-1) to D(8) in HVD regions for all of the treated mice.

For all of the sunitinib-treated tumors, D(-1:3) LVD ∆HbT metric showed good correlation with both pre-treatment LVD Fraction (Figure 9D, r = -0.578, p-value < 0.01) and pre-treatment HbT (Figure 9E, r = -0.648, p-value < 0.0001. Pre-treatment StO_2_ displayed a strong negative correlation with the LVD ∆StO_2_ from D(-1) to D(3) (Figure 9F, r = -0.762, p-value <0.0001), indicating that tumors with higher pre-treatment StO_2_, more LVD areas, and higher average HbT tended to undergo a more drastic reduction in oxygenation and HbT content in LVD regions in response to sunitinib.

Interestingly, we observe that the D(-1:8) HVD metrics also displayed a negative relationship with pre-treatment LVD Fraction (Figure 9G, r = -0.795, p-value <0.00001), HbT (Figure 9H, r = -0.700, p-value < 0.0001), and StO_2_ (Figure 9I, r = -0.562, p-value < 0.01), showing that the tumors with the lowest pre-treatment levels of these metrics showed the largest increase of blood content and oxygen in HVD regions at D(8). From this, we can infer that the level of vascular remodeling occurring from D(-1:8) may directly relate to the initial level of vascular pruning from D(-1:3). We correlated both D(-1:3) LVD ∆StO_2_ with D(-1:8) HVD ∆StO_2_ and D(-1:3) LVD ∆HbT with D(-1:8) HVD ∆HbT to further understand the relationship between vascular pruning and vessel normalization in treated tumors. Interestingly when correlating the D(-1:3) LVD with D(-1:8) HVD metrics, the levels of microvascular pruning (D(-1:3) LVD ∆HbT) effects the change in functional vessel blood content (D(-1:8) HVD ∆HbT) more drastically (r = 0.557, p-value < 0.01) than the levels of microvascular deoxygenation (D(-1:3) LVD ∆StO_2_) effect the D(-1:8) HVD ∆StO_2_ transient reoxygenation (r = 0.430, p-value < 0.05). These positive relationships align with many reports of vascular normalization where tumors that do not undergo complete vascular annihilation are shown to develop more mature and functional vasculature 19, 24, 25, 51. Moreover, these results also illuminate the need for more extensive studies needed to understand the role of pre-treatment hemodynamics on tumor response to anti-vascular therapies, and thereby personalize the treatment strategy for effective outcomes.

## Discussion and Conclusion

Comprehensive knowledge of the microvascular alterations within a tumor in response to anti-angiogenic therapy is crucial for optimizing efficacy and predicting long-term effects. Several methodologies are currently employed for microvessel segmentation in endogenous contrast PAI such as threshold-based segmentation 96, 124, morphology-based segmentation 92, 93, 98, 125, 126, and deep learning-based segmentation 95, 99, 127, 128. While these methodologies can directly measure MVD based on microvessel segmentation, they have only been applied to microscopic or mesoscopic imaging configurations 92-100, 124-129 that provide high resolution but lack sufficient penetration depth to enable 3D visualization in a clinical setting. This reiterates the need to develop vascular segmentation or classification methodologies which can be applied to macroscopic, relatively low-resolution photoacoustic images. To this end, we successfully developed a methodology for VRA, which is specifically applicable to *in vivo* macroscopic 3D PA images. The VRA methodology is able to differentiate LVD and HVD areas within a tumor to serve as surrogate markers for relative MVD. The VRA methodology was validated with quantitative histological analysis which revealed significant positive correlation between regions labeled HVD and endothelial marker CD31 (r = 0.695). Once the reliability of VRA was confirmed, we investigated the feasibility of utilizing VRA for monitoring therapy response in solid tumors.

The segmentation methodology was utilized to perform regional analysis on murine pancreatic xenografts treated with the TKI sunitinib. Our findings show that sunitinib induced significant changes in the oxygenation of both AsPC-1 and MIA PaCa-2 xenografts within the first 72 h of the treatment regimen in agreement with previous literature investigating the effects of anti-angiogenic therapy on subcutaneous pancreatic xenografts 82. Region-based analysis indicated that sunitinib preferentially reduced StO₂ in LVD regions in AsPC-1 tumors but not MIA PaCa-2 tumors. At 24 h and 72 h post-treatment, StO₂ was significantly different between treated and control tumors in only LVD regions for AsPC-1. At these junctures, we also observed significant differences in ∆StO₂ when comparing HVD and LVD regions within treated tumors that were not present in untreated tumors. This indicates that the discrepancies seen are due to preferential targeting by sunitinib rather than non-treatment effects. The administration of sunitinib to AsPC-1 xenografts resulted in transient deoxygenation and reduction in vessel density that occurred preferentially within LVD regions. This was followed by reoxygenation in all regions and an increase in blood volume in HVD regions between D(3) and D(8), indicating that the remaining blood vessels had improved functionality within the reoxygenation window. This was further proven through triple sequential staining of endothelial cells, pericytes, and perfusing vessels using CD31, aSMA, and TL, where treated AsPC-1 tumors displayed increased vascular maturity and perfusion compared to non-treated tumors on D(8). On the other hand, these same metrics showed no conclusive evidence of vascular normalization in MIA PaCa-2 tumors.

The impact of anti-angiogenic therapies on tumor StO₂ is a subject of ongoing debate. Several studies have shown sunitinib therapy to promote vascular normalization by reorganizing the growth of new blood vessels in tumors by removing dysfunctional vessels 32, 59, 66, 69. This vascular remodeling can promote the reoxygenation of tumors, showing promise for combination with treatments such as radiation or photodynamic therapy, which require oxygen 130, 131. Conversely, other studies have found that sunitinib treatment does not restore normal blood vessel structure and instead leads to hypoxia 49, 67. Sunitinib-induced VEGFR inhibition reduces pathological vascular proliferation and enhances the structural integrity of blood vessels, while PDGFR inhibition decreases pericyte recruitment, which can initially exacerbate vessel dysfunction, leading to hypoxia 50, 63. However, when vessels are partially normalized through the controlled application of these inhibitors, a more optimal pericyte coverage can occur, leading to improvements in vessel stability, permeability, and perfusion 70, 71 as displayed in the AsPC-1 tumors. This homeostatic balance between endothelial cells and pericytes enhances oxygen and nutrient delivery to tumor tissues, as shown through VRA by the increased HVD StO₂ and HbT during the identified vascular normalization window.

The baseline vascular characteristics of the different tumor models hint towards the differential response of AsPC-1 to sunitinib compared to MIA PaCa-2. One of the most crucial pro-angiogenic factors in cancer is VEGF, which has two primary roles mediated by the kinase insert domain receptor (KDR) gene: promoting the growth of new blood vessels (angiogenesis) and increasing the permeability of blood vessels (vascular hyperpermeability) 132-134. The gene effect scores for KDR and KIT are negative in both cell lines and lower in AsPC-1 (KDR: -0.300, KIT: -0.103) than MIA PaCa-2 (KDR: -0.231, KIT: -0.056), indicating higher dependency of VEGFR and KIT for cell growth in AsPC-1^135^, ^136^, which could explain the finding that MIA PaCa-2 tumors display slightly poorer volumetric response compared to AsPC-1. The gene effect scores could not be found for BxPC-3 cell line. The observation that LVD fractions were significantly lower, and HbT was significantly higher in MIA PaCa-2 before treatment onset aligns with additional *in vitro* work showing higher expression of pro-angiogenic factors COX-2 137 and VEGF 138 when compared directly with AsPC-1. Our results combined with the aforementioned *in vitro* work strongly imply that AsPC-1 tumors may promote less angiogenic activity and contain more immature microvasculature than MIA PaCa-2.

Selective destruction of immature blood vessels from anti-angiogenic therapy is a known phenomenon 87, 139, and preferential targeting of sunitinib displayed in AsPC-1 and MIA PaCa-2 could be due to the relative maturity of the microvessels in these regions. Investigation of the mechanisms behind the favorable targeting of specific vascular regions by sunitinib requires in-depth analysis that is beyond the scope of this work, but an important future direction. Quantitative assays that measure the level of vascular maturation pre-treatment could potentially indicate the vulnerability of the tumor's existing blood vessels to sunitinib and would be a logical next step in this work. One weakness of this study is that a subcutaneous model was used, which may not accurately reflect the biology of human PC as much as a genetically engineered mouse model, or orthotopic implantation 140, 141.

Overall, our study demonstrates the feasibility of using VRA in macroscopic US-PAI to monitor the vascular microenvironmental changes caused by TKI therapy. VRA coupled with macroscopic US-PAI has the potential to provide valuable insights for the evaluation of key time points in which anti-angiogenic therapy is promoting vascular normalization, and how the intratumoral vascular density affects this progression. An extensive amount of further study is needed in dose optimization of anti-vascular therapies as those that induce hypoxia may render tumors less responsive to the majority of conventional therapies, adding an obstacle to the successful implementation of combination therapies. The present research did not include any experiments that combined sunitinib treatment with radiation therapy or chemotherapy. Nevertheless, the differences between HVD and LVD areas highlight the importance of accounting for relative vascular density in measurements of tumor StO₂ and HbT, and the potential of VRA to provide additional prognostic markers of treatment response and to identify crucial time points in which angiogenesis inhibitors can work synergistically with traditional therapeutics, particularly for tumors with low pro-angiogenic potential.

## Supplementary Material

Supplementary figures and tables.

## Abbreviations

σ: standard deviation
∆x̄: difference between means of two groups
aSMA: alpha smooth muscle actin
Ab: antibody
AV: avascular
CD31: cluster of differentiation 31
D(#): day #
DAPI: 4',6-diamidino-2-phenylindole
DMEM: Dulbecco's modified eagle medium
ECG: electrocardiogram
FFT: fast Fourier transform
GIST: gastrointestinal stromal tumor
H&E: Hematoxylin and Eosin
Hb: deoxyhemoglobin
HbO2: oxyhemoglobin
HbT: total hemoglobin content
HVD: high vascular density
IF: immunofluorescence
IFFT: inverse fast Fourier transform
KDR: kinase insert domain receptor
KIT: cluster of differentiation 117
LN: log-normal
LVD: low vascular density
MCX: Monte Carlo extreme
MVD: microvessel density
NIR: near infrared
NT: no treatment
PA: photoacoustic(s)
PAI: photoacoustic imaging
PBS: phosphate buffered saline
PC: pancreatic cancer
PDGF(R): platelet-derived growth factor (receptor)
PHANTOM: photoacoustic annotation toolkit for MATLAB
ROI: region of interest
RPMI-1640: Roswell Park Memorial Institute
SEM: standard error of the mean
SNR: signal-to-noise ratio
StO₂: blood oxygen saturation
TKI: tyrosine kinase inhibitor
TL: tomato lectin
TME: tumor microenvironment
US: ultrasound
US-PAI: ultrasound-guided photoacoustic imaging
VEGF(R): vascular endothelial growth factor (receptor)
VNI: vascular normalization index
VRA: vascular regional analysis
