# Supplementary material for: Vascular regional analysis unveils differential responses to anti-angiogenic therapy in pancreatic xenografts through macroscopic photoacoustic imaging
Source: Theranostics. 2025 Jan 27;15(6):2649–71. doi: 10.7150/thno.99361 (PMC11840746; doi:10.7150/thno.99361)
Supplement: Supplementary file 1 — Supplementary figures and tables. [file thnov15p2649s1.pdf]

Vascular regional analysis unveils differential responses to anti-angiogenic therapy in pancreatic xenografts through macroscopic photoacoustic imaging

Allison Sweeney[1], Andrew Langley[1], Marvin Xavierselvan[1], Ronak Shethia[1], Patrick Solomon[1], Aayush Arora[1], and Srivalleesha Mallidi[1,2 *]

[1]Department of Biomedical Engineering, Tufts University, Medford, MA, United States
[2] Wellman Center for Photomedicine, Massachusetts General Hospital, Boston, MA, United States

* Corresponding author: Srivalleesha Mallidi, Department of Biomedical Engineering, Tufts University, Medford MA 02155, srivalleesha.mallidi@tufts.edu

Keywords: pancreatic cancer, angiogenesis, sunitinib, vascular density, photoacoustics, endogenous contrast, vascular normalization

Tissue	μ_a (mm^{-1})<br>750 nm | 850 nm	μ_s (mm^{-1})<br>750 nm | 850 nm	Anisotropy	Refractive Index
Skin	0.2504 | 0.2935	25.22 | 21.90	0.9	1.37
Tumor	0.0051 | 0.0061	5.278 | 4.578	0.9	1.37
Standard Tissue	0.0110 | 0.0199	10.97 | 9.418	0.9	1.37

Table S1. Optical properties used to fluence compensate PA scans of subcutaneous tumors.

Day	Mean Threshold	Standard Deviation	N
-1	0.00073	1.1e-005	55
1	0.00075	1.6e-005	55
3	0.00083	2.1e-005	55
6	0.00080	2.8e-005	48
8	0.00070	1.7e-005	47
11	0.00074	3.2e-005	30
13	0.00071	1.8e-005	29
15	0.00072	2.9e-005	28
18	0.00078	6.5e-005	26
20	0.00071	2.5e-005	17
ALL	0.00075	4.2e-005	390

Table S2. Calculated threshold for all mice on a particular day, with standard deviation, and sample size (N)

Tumor	HVD vs CD31 amplitude
1	1.75e-06
2	1.98e-07
3	6.58e-09
en mass	1.16e-19

Table S3. P-values for Pearson's r used in correlation analysis between CD31 and fraction of HVD. Tumor numbers correspond to those shown in Fig S1.

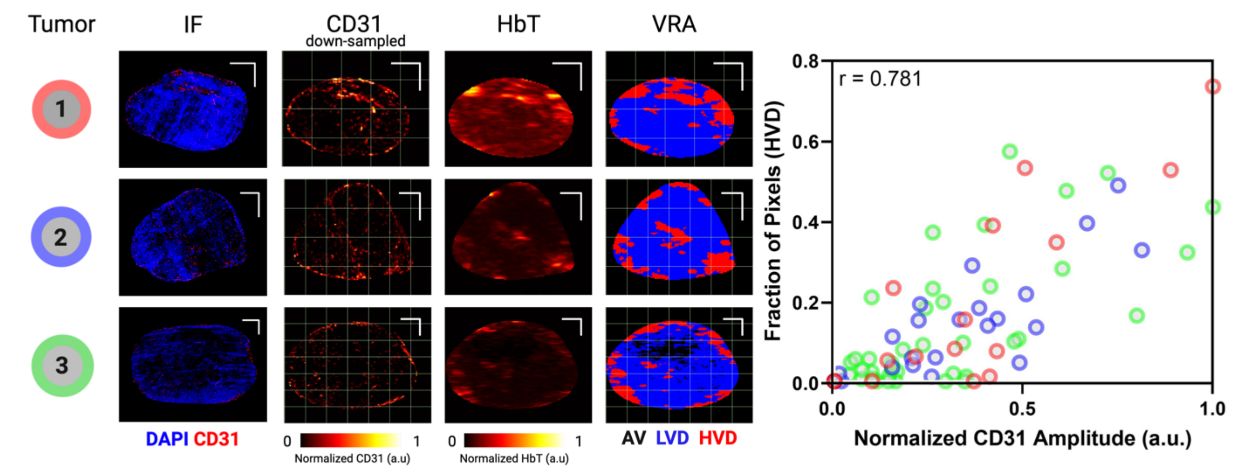

Figure S1. Individual tumor cross-sections used to validate VRA algorithm. First image column shows the pre-processed immunofluorescence (IF) image with CD31 displayed in red and the DAPI counterstain displayed in blue. Second column displays the CD31 stain after down-sampling, overlayed with the 1 mm x 1 mm ROIs used for correlation analysis. The third image column shows the matching HbT cross-section input into the vascular regional analysis (VRA) algorithm. The output of the VRA algorithm is shown in the right-most image column and shows areas labeled as high vascular density (HVD) in red, low vascular density (LVD) in blue, and avascular (AV) regions in black. The VRA image is overlayed with the same ROIs used for correlation analysis as shown in the second column of images. All image scale bars represent 1 mm in the x- and y-direction. The average CD31 amplitude is plotted against the fraction of pixels labeled HVD for each 1 mm x 1 mm ROI shown in the down-sampled CD31 images and the VRA images for each tumor.

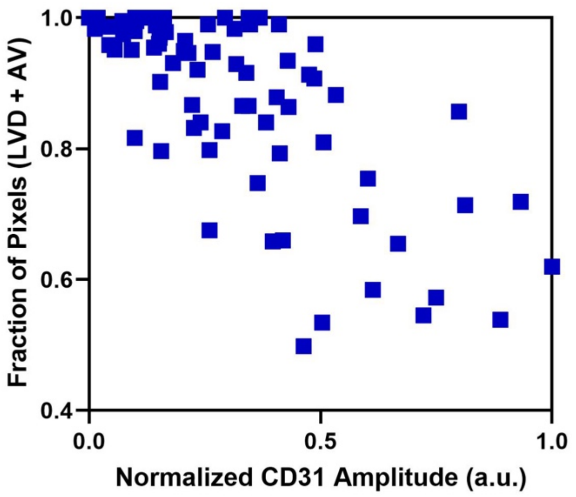

Figure S2. Plot of normalized average CD31 intensity in a 1 mm x 1 mm ROI versus the fraction of pixels in the ROI labelled as LVD or AV.

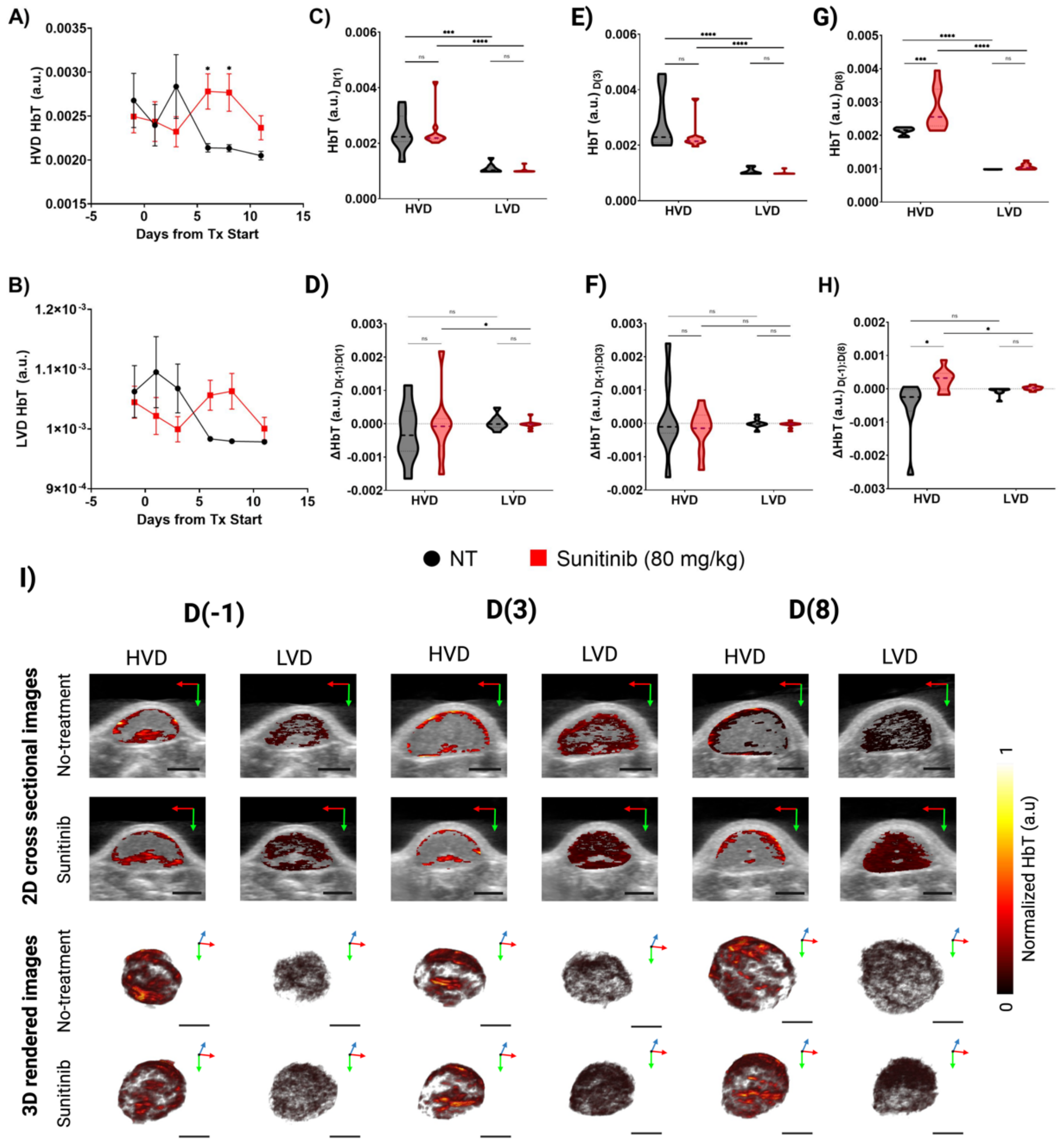

Figure S3. A) Plot of HbT in sunitinib (80 mg/kg) treated (red line) and untreated control (no treatment, black line) AsPC-1 tumors in regions of high vascular density (HVD). **B)** Plot of HbT in sunitinib at 80 mg/kg (red line), and no treatment (black line) AsPC-1 tumors in low vascular density (LVD) regions. **C)** Violin plot comparing HbT on D(1) for Sunitinib at 80 mg/kg (red) and No Treatment (black) AsPC-1 tumors in areas HVD and LVD. **D)** Violin plot comparing ΔHbT between D(1) and D(-1) for sunitinib at 80 mg/kg (red) and no treatment (black) AsPC-1 tumors in areas of HVD and LVD. **E)** Violin plot comparing HbT on D(3) for Sunitinib at 80 mg/kg (red) and No Treatment (black) AsPC-1 tumors in areas HVD and LVD. **F)** Violin plot comparing ΔHbT between D(3) and D(-1) for sunitinib at 80 mg/kg (red) and no treatment (black) AsPC-1 tumors in areas of HVD and LVD. **G)** Violin plot comparing HbT on D(8) for Sunitinib at 80 mg/kg (red) and No Treatment (black) AsPC-1 tumors in areas HVD and LVD. **H)** Violin plot comparing ΔHbT between D(8) and D(-1) for sunitinib at 80 mg/kg (red) and no treatment (black) AsPC-1 tumors in areas of HVD and LVD. **I)** Regional 2D cross sectional images and 3D rendered images of HbT in Sunitinib (80 mg/kg) and No Treatment tumors displaying high vascular density areas (HVD), and low vascular density areas (LVD). These HbT images correspond to the StO_2 images shown for AsPC-1 in Fig 6.

All error bars shown represent SEM. p-values: * < 0.05, ** < 0.01, *** < 0.001, # < 0.0001, ## < 0.00001

Scale bars = 2 mm

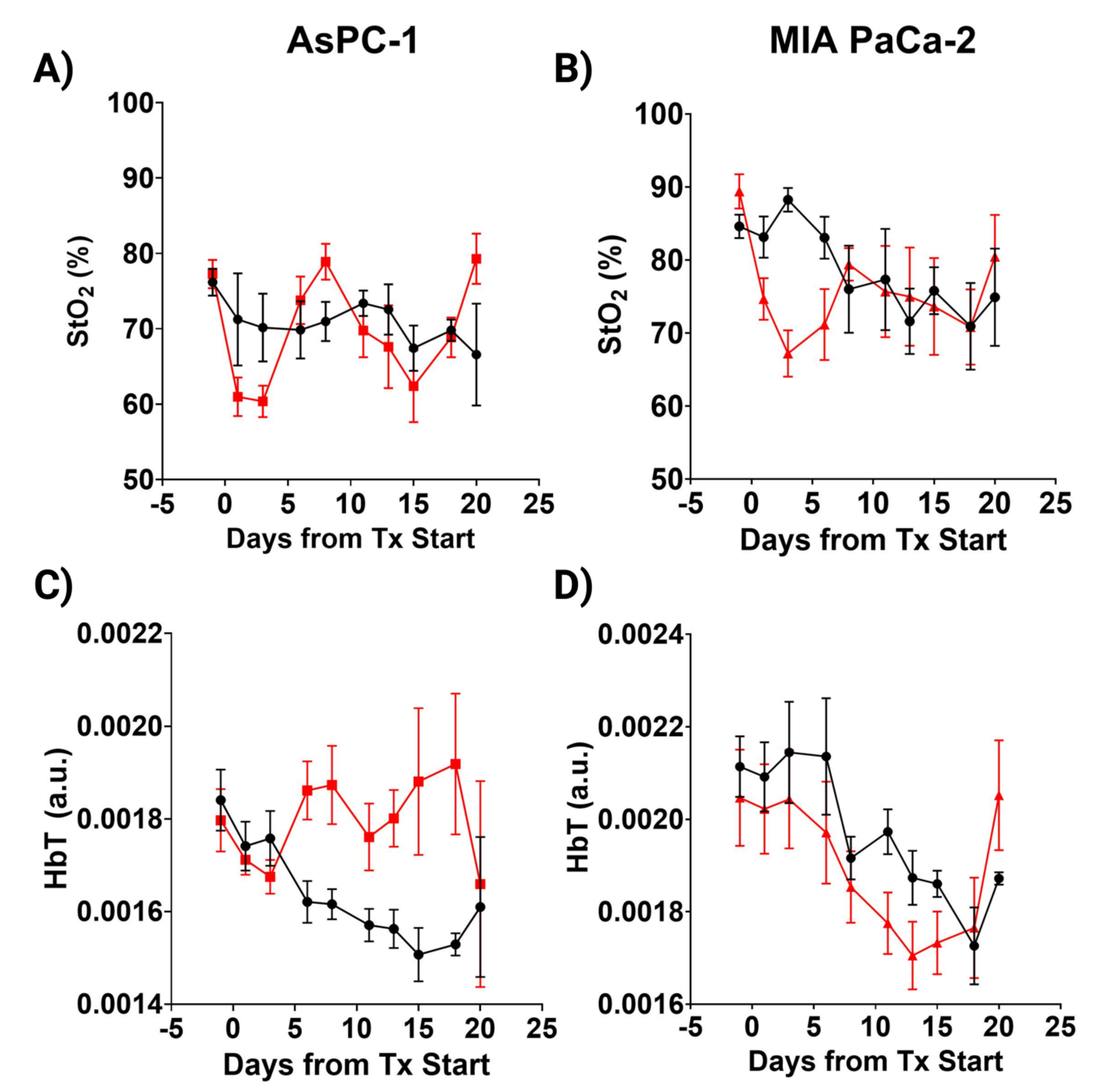

Figure S4. Profiles of StO2 and HbT in sunitinib (80 mg/kg) treated (red line) and untreated control (no treatment, black line) in the whole AsPC-1 (**A, C**) and MIA PaCa-2 (**B,D**) tumors.

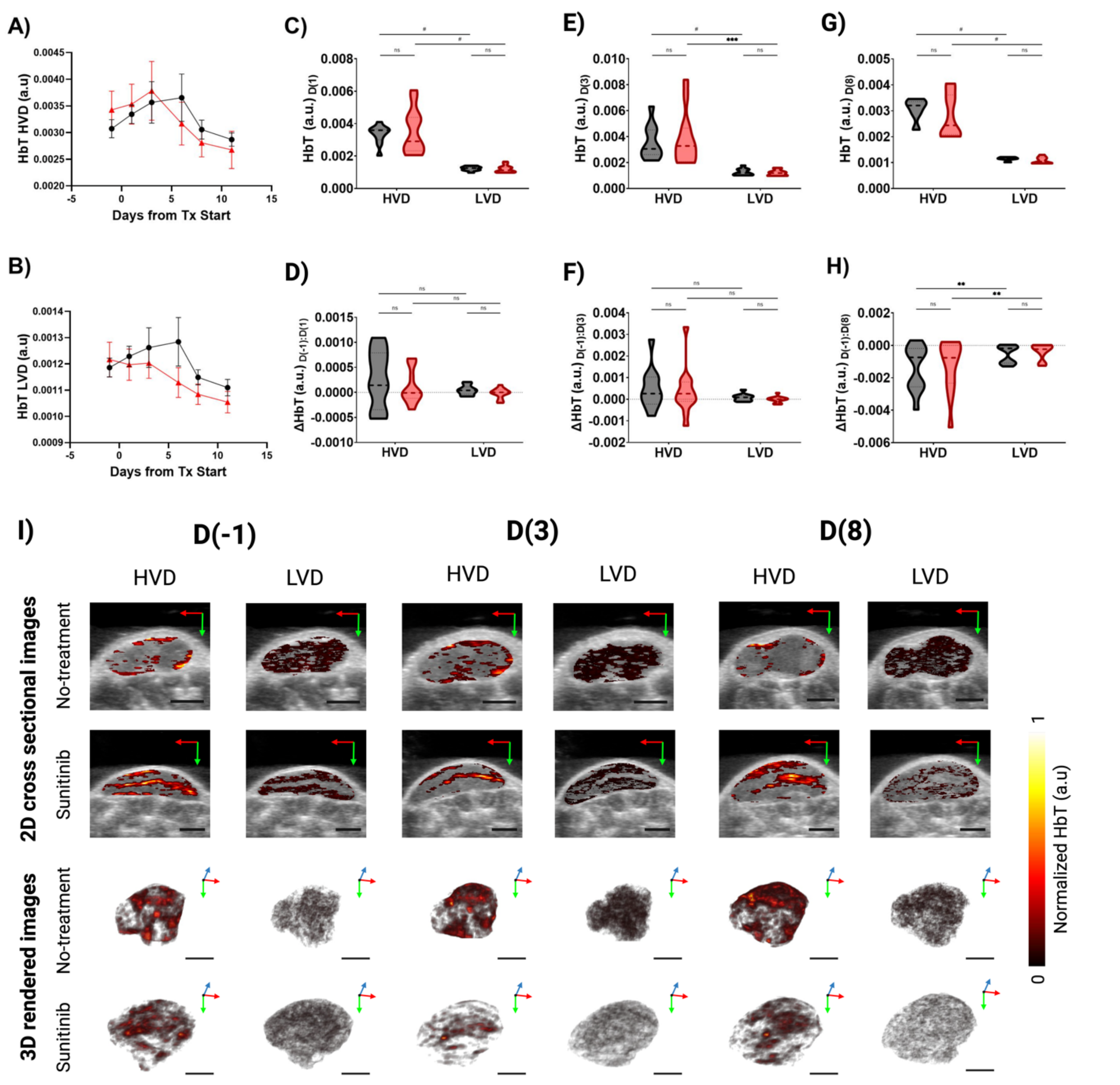

Figure S5. A) Plot of HbT in sunitinib (80 mg/kg) treated (red line) and untreated control (no treatment, black line) MIA PaCa-2 tumors in regions of high vascular density (HVD). **B)** Plot of HbT in sunitinib at 80 mg/kg (red line), and no treatment (black line) MIA PaCa-2 tumors in low vascular density (LVD) regions. **C)** Violin plot comparing HbT on D(1) for Sunitinib at 80 mg/kg (red) and No Treatment (black) MIA PaCa-2 tumors in areas HVD and LVD. **D)** Violin plot comparing ΔHbT between D(1) and D(-1) for sunitinib at 80 mg/kg (red) and no treatment (black) MIA PaCa-2 tumors in areas of HVD and LVD. **E)** Violin plot comparing HbT on D(3) for Sunitinib at 80 mg/kg (red) and No Treatment (black) MIA PaCa-2 tumors in areas HVD and LVD. **F)** Violin plot comparing ΔHbT between D(3) and D(-1) for sunitinib at 80 mg/kg (red) and no treatment (black) MIA PaCa-2 tumors in areas of HVD and LVD. **G)** Violin plot comparing HbT on D(8) for Sunitinib at 80 mg/kg (red) and No Treatment (black) MIA PaCa-2 tumors in areas HVD and LVD. **H)** Violin plot comparing ΔHbT between D(8) and D(-1) for sunitinib at 80 mg/kg (red) and no treatment (black) MIA PaCa-2 tumors in areas of HVD and LVD **I)** Regional 2D cross sectional images and 3D rendered images ofvHbT in Sunitinib (80 mg/kg) and No Treatment tumors displaying high vascular density areas (HVD), and low vascular density areas (LVD). These HbT images correspond to the StO_2 images shown for MIA-PaCa-2 in Fig 7.

All error bars shown represent SEM. p-values: * < 0.05, ** < 0.01, *** < 0.001, # < 0.0001, ## < 0.00001

Scale bars = 2 mm

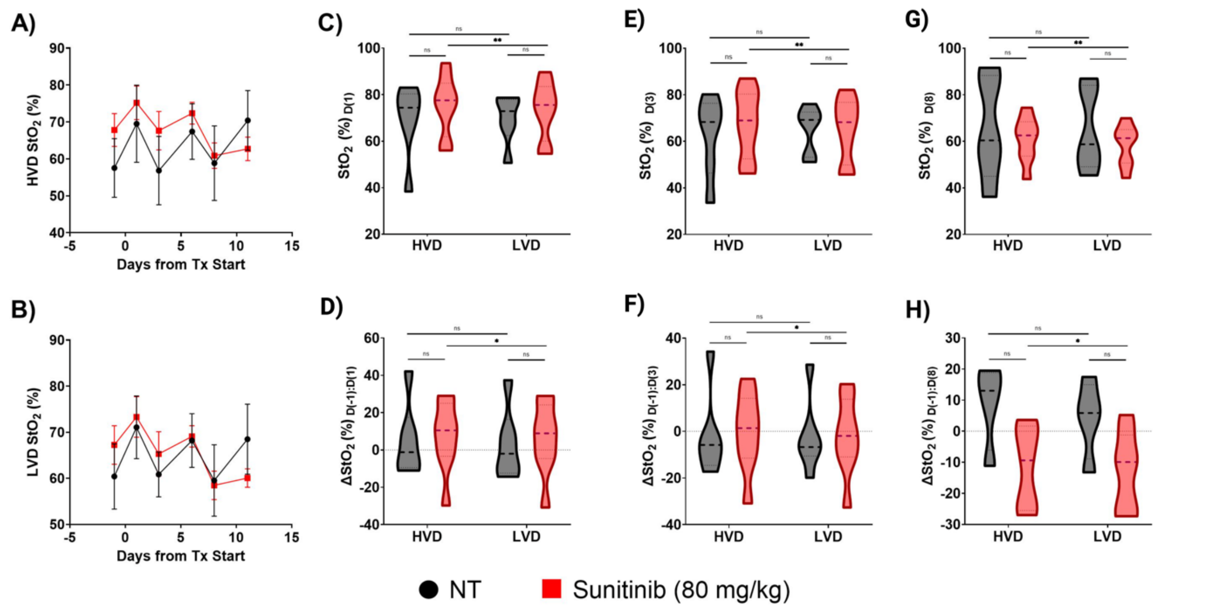

Figure S6. Profiles of StO_2 in sunitinib (80 mg/kg) treated (red line) and untreated control (no treatment, black line) BxPC-3 tumors in regions of (**A**) high vascular density (HVD). and (**B**) low vascular density (LVD) regions. **C)** Violin plot comparing StO_2 on D(1) for Sunitinib at 80 mg/kg (red) and No Treatment (black) BxPC-3 tumors in areas HVD and LVD. **D)** Violin plot comparing ΔStO_2 between D(1) and D(-1) for sunitinib at 80 mg/kg (red) and no treatment (black) BxPC-3 tumors in areas of HVD and LVD. **E)** Violin plot comparing StO_2 on D(3) for Sunitinib at 80 mg/kg (red) and No Treatment (black) BxPC-3 tumors in areas HVD and LVD. **F)** Violin plot comparing ΔStO_2 between D(3) and D(-1) for sunitinib at 80 mg/kg (red) and no treatment (black) BxPC-3 tumors in areas of HVD and LVD. **G)** Violin plot comparing StO_2 on D(8) for Sunitinib at 80 mg/kg (red) and No Treatment (black) BxPC-3 tumors in areas HVD and LVD. **H)** Violin plot comparing ΔStO_2 between D(8) and D(-1) for sunitinib at 80 mg/kg (red) and no treatment (black) BxPC-3 tumors in areas of HVD and LVD. All error bars shown represent SEM. p-values: * < 0.05, ** < 0.01, *** < 0.001, # < 0.0001, ## < 0.00001

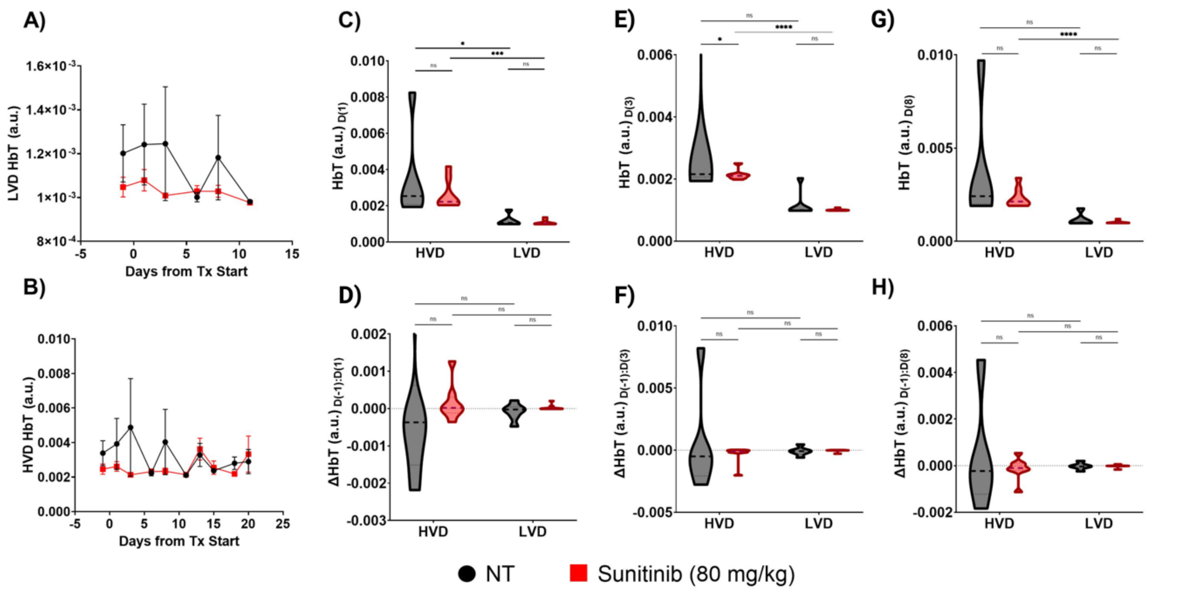

Figure S7. Profiles of HbT in sunitinib (80 mg/kg) treated (red line) and untreated control (no treatment, black line) BxPC-3 tumors in regions of (**A**) high vascular density (HVD) and (**B**) low vascular density (LVD) regions. **C)** Violin plot comparing HbT on D(1) for Sunitinib at 80 mg/kg (red) and No Treatment (black) BxPC-3 tumors in areas HVD and LVD. **D)** Violin plot comparing ΔHbT between D(1) and D(-1) for sunitinib at 80 mg/kg (red) and no treatment (black) BxPC-3 tumors in areas of HVD and LVD. **E)** Violin plot comparing HbT on D(3) for Sunitinib at 80 mg/kg (red) and No Treatment (black) BxPC-3 tumors in areas HVD and LVD. **F)** Violin plot comparing ΔHbT between D(3) and D(-1) for sunitinib at 80 mg/kg (red) and no treatment (black) BxPC-3 tumors in areas of HVD and LVD. **G)** Violin plot comparing HbT on D(8) for Sunitinib at 80 mg/kg (red) and No Treatment (black) BxPC-3 tumors in areas HVD and LVD. **H)** Violin plot comparing ΔHbT between D(8) and D(-1) for sunitinib at 80 mg/kg (red) and no treatment (black) BxPC-3 tumors in areas of HVD and LVD. All error bars shown represent SEM. p-values: * < 0.05, ** < 0.01, *** < 0.001, # < 0.0001, ## < 0.00001
